# Mitochondrial DNA barcoding of mosquito species (Diptera: Culicidae) in Thailand

**DOI:** 10.1371/journal.pone.0275090

**Published:** 2022-09-22

**Authors:** Tanawat Chaiphongpachara, Tanasak Changbunjong, Sedthapong Laojun, Teerayoot Nutepsu, Nantana Suwandittakul, Kewarin Kuntawong, Suchada Sumruayphol, Jiraporn Ruangsittichai

**Affiliations:** 1 Department of Public Health and Health Promotion, College of Allied Health Sciences, Suan Sunandha Rajabhat University, Bangkok, Thailand; 2 Faculty of Veterinary Science, Department of Pre-Clinic and Applied Animal Science, Mahidol University, Nakhon Pathom, Thailand; 3 Faculty of Veterinary Science, The Monitoring and Surveillance Center for Zoonotic Diseases in Wildlife and Exotic Animals (MoZWE), Mahidol University, Nakhon Pathom, Thailand; 4 Faculty of Tropical Medicine, Department of Medical Entomology, Mahidol University, Bangkok, Thailand; Sichuan University, CHINA

## Abstract

The correct identification of mosquito species is important for effective mosquito vector control. However, the standard morphological identification of mosquito species based on the available keys is not easy with specimens in the field due to missing or damaged morphological features during mosquito collections, often leading to the misidentification of morphologically indistinguishable. To resolve this problem, we collected mosquito species across Thailand to gather genetic information, and evaluated the DNA barcoding efficacy for mosquito species identification in Thailand. A total of 310 mosquito samples, representing 73 mosquito species, were amplified using mitochondrial cytochrome *c* oxidase subunit I (*COI*) primers. The average maximum intraspecific genetic variation of the 73 mosquito species was 1% ranged from 0–5.7%. While, average minimum interspecific genetic variation (the distance to the nearest neighbour) of the 73 mosquito species was 7% ranged from 0.3–12.9%. The identification of success rates based on the “Best Match,” “Best Close Match,” and “All Species Barcodes” methods were 97.7%, 91.6%, and 81%, respectively. Phylogenetic analyses of *Anopheles COI* sequences demonstrated a clear separation between almost all species (except for those between *An*. *baimaii* and *An*. *dirus*), with high bootstrap support values (97%–99%). Furthermore, phylogenetic analyses revealed potential sibling species of *An*. *annularis*, *An*. *tessellatus*, and *An*. *subpictus* in Thailand. Our results indicated that DNA barcoding is an effective molecular approach for the accurate identification of mosquitoes in Thailand.

## Introduction

Mosquitoes are small flying insects that belong to the order Diptera (two-winged flies), and the family Culicidae. Currently, 3,611 species of mosquitoes are formally recognized, which can be classified into two subfamilies and 113 genera, with numerous further species awaiting confirmation [1]. Several mosquito species are important from the perspective of tropical medicine and public health, because they are vectors for infectious pathogens, including nematodes, protozoans, and arboviruses, that cause dangerous diseases in humans [2]. Mosquito-borne infectious diseases are considered public health threats in several countries, especially those located in tropical and temperate regions [3]. The World Health Organization estimated that in humans, vector-borne diseases account for >17% of all infectious diseases and 700,000 deaths annually [4]. Thailand is endemic to mosquito-borne diseases, such as dengue, chikungunya, Zika, malaria, and Japanese encephalitis [5]. Annually, >100,000 cases and >100 deaths occur in Thailand due to mosquito-borne diseases [6].

The epidemiology of mosquito-borne infectious diseases is strongly related to the mosquito species. Each mosquito species exhibits biological and ecological differences related to their resting and biting behavior, vectorial capacity, and habitat [7,8]. Therefore, a clear identification of the mosquito species is essential for developing species-specific vector controls. The correct identification of the mosquito species helps provide accurate vector information, leading to the effective control of mosquito-borne diseases [7,9]. However, the standard morphological identification of mosquito species based on the available keys is not easy with specimens in the field due to missing or damaged morphological features during mosquito collections, often leading to the misidentification of morphologically indistinguishable [10]. Furthermore, several *Anopheles* mosquito species form complex groups, comprising morphologically indistinguishable sibling species [11]. Our knowledge in molecular biology has undergone significant progress, leading to the development of techniques useful for species identification [12]. Therefore, the use of modern molecular biology techniques for the species-based identification of mosquito vectors is an extremely attractive alternative to standard morphological methods [13].

DNA barcoding is a molecular biological approach that has gained wide attention because of its efficiency and accuracy in identifying species of mammals [14], birds [15], reptiles, amphibians [16], fishes [17], and arthropods [18]. This diagnostic molecular assay is based on the principle that each animal species displays its own unique set of short DNA fragments that differ among species, similar to the barcodes of items in a department store that show information about the items [19,20]. DNA barcoding relies on amplifying a highly conserved and standardized short region of DNA (approximately 400–800 base pairs [bp]) using PCR for species-level taxonomy [21]. Mitochondrial cytochrome *c* oxidase subunit I (*COI*), a conserved gene and the first standard genetic region used for animal DNA barcoding [22], is the most popular barcode marker. However, this technique requires reliable reference sequences stored in international DNA barcoding libraries to identify unknown species. This often represents a significant problem with the absence of nucleotide sequences of a given species in the database for genetic comparison or the presence of insufficient sequences; leading to a lack of credibility [23,24].

In medical entomology, DNA barcoding is used to confirm insect species that are difficult to identify by morphological methods, such as black flies [25], biting midges [26], sand flies [27], horse flies [28], deer flies [29], flesh flies [30], and mosquitoes [31–34]. Mosquitoes have been widely and successfully investigated by this molecular technique in various countries [23,35–38]. However, this technique might not be efficient in distinguishing certain mosquito species because of insufficient differences in their mitochondrial nucleotide sequences, such as for *Anopheles deaneorum* and *Anopheles marajoara* [39] or *Anopheles albertoi* and *Anopheles strodei* [40]. Although several studies report using DNA barcoding to solve the problem of identifying mosquito vectors in Thailand, most focus only on the main mosquito vectors. To address these problems, we collected mosquito species across Thailand to gather genetic information, and submitted it to an international reference database for facilitating taxonomic studies in mosquitoes. In addition, we analyzed mitochondrial *COI* sequence to evaluate the DNA barcoding efficacy for mosquito species identification in Thailand.

## Materials and methods

### Ethics statement

This study was strictly conducted according to the animal care and use guidelines of the Suan Sunandha Rajabhat University, Thailand. Animal care and experimental procedures were reviewed and approved by the Institutional Animal Care and Use Committee of the Suan Sunandha Rajabhat University, Bangkok, Thailand (Animal Ethics Permission number: IACUC 64-004/2021).

### Study sites and mosquito collection

**Fig 1:**
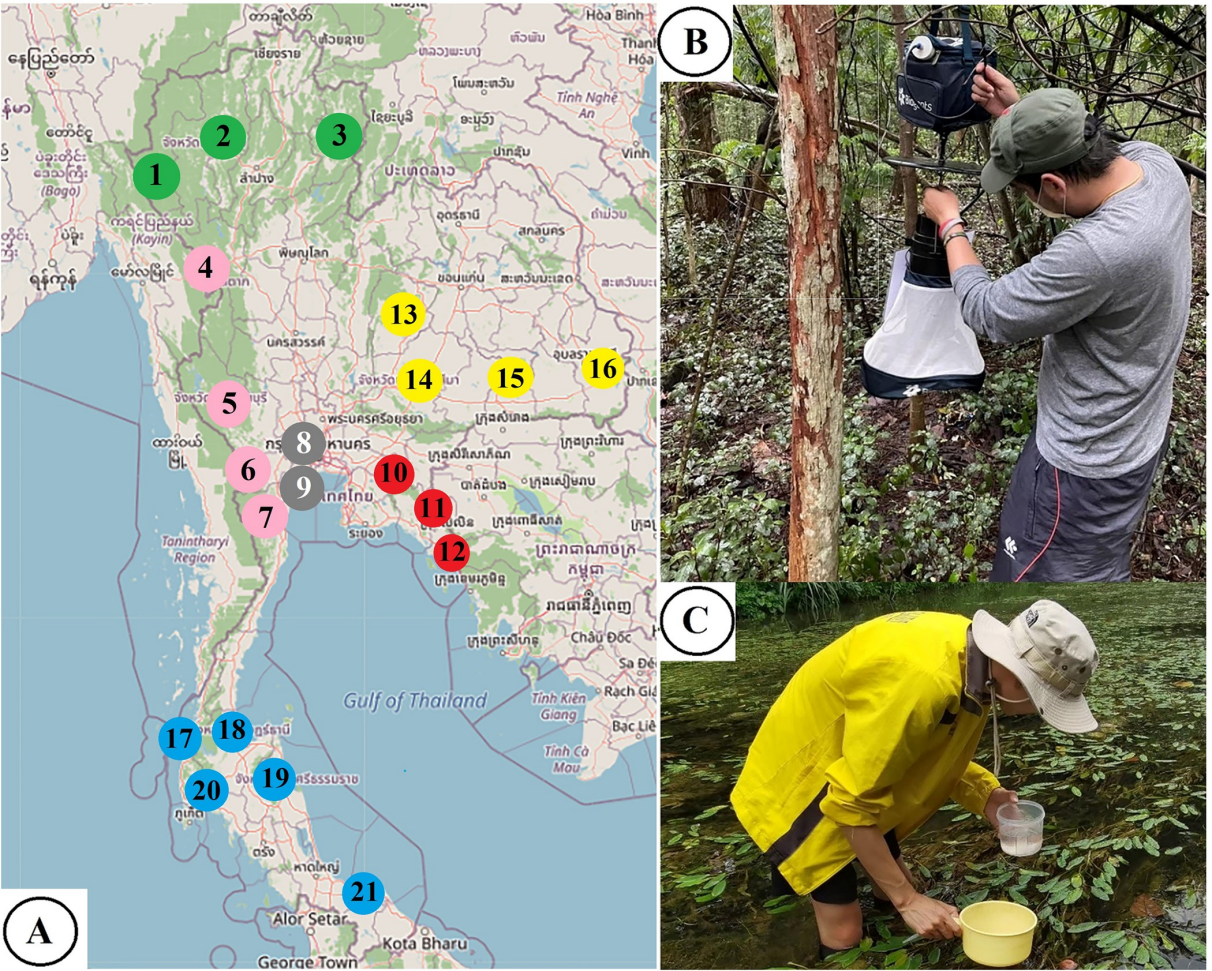
Map of the study sites (A) and mosquito-collection methods used, including adult mosquito trapping (B) and larvae dipper (C). Mosquito samples were collected from 22 provinces from six geographic regions of Thailand, comprising the Northern region (green), including (1) Mae Hong Son, (2) Chiang Mai, and (3) Nan; the Western region (pink) including (4) Tak, (5) Kanchanaburi, (6) Ratchaburi, and (7) Phetchaburi; the Central region (gray), including (8) Nakhon Pathom and (9) Samut Songkhram; the Eastern region (red), including (10) Chachoengsao, (11) Chanthaburi, and (12) Trat; the Northeastern region (yellow), including (13) Chaiyaphum, (14) Nakhon Ratchasima, (15) Surin, and (16) Ubon Ratchathani; and the Southern region (blue), including (17) Phang Nga, (18) Surat Thani, (19) Nakhon Si Thammarat, (20) Krabi, and (21) Narathiwat. Free map provided by USGS National Map Viewer (public domain): http://viewer.nationalmap.gov/viewer/.

Mosquito samples were collected from all regions of Thailand in 2021. The sites for mosquito collections in each province were selected based on the reports on endemic areas of mosquito-borne infectious diseases from Thailand’s National Disease Surveillance (available at http://doe.moph.go.th/surdata/index.php) (Fig 1A). To ensure species richness of the samples, two mosquito-collection methods, including adult mosquito trapping and larvae dipper, were used (Fig 1B and 1C). Adult mosquitoes were captured using the BG-Pro CDC-style mosquito trap (BioGents, Regensburg, Germany), with the BG-lure cartridge (BioGents, Regensburg, Germany) and solid carbon dioxide (dry ice) at night between 6:00 pm and 6:00 am. Mosquito traps were hung above the ground at approximately 1.5 and 10 m of residential houses. Mosquito larvae were collected using plastic dippers from various water sources at the study site. The collected larvae were placed in plastic trays and their collection dates and sources were labeled on the lids. The samples were transported to the laboratory at the College of Allied Health Sciences, Suan Sunandha Rajabhat University, Thailand. The larvae were reared in white plastic trays (25 × 30 × 5 cm) under laboratory conditions (25°C–28°C, a 12–12-h light–dark cycle, and 50%–60% relative humidity). At the pupal stage, they were transferred into mosquito cages (30 × 30 × 30 cm) to facilitate adult stage emergence. All adult female mosquitoes, identified based on their morphological features under a stereomicroscope using several standard taxonomic keys, were killed in the freezer at −20°C [41–47]. Then, the mosquito samples identified were placed in 1.5-mL microcentrifuge tubes (one sample per tube) with 95% ethanol and stored in the freezer at −20°C until DNA extraction.

### DNA extraction, PCR amplification, and sequencing

Total genomic DNA was extracted from 2 to 4 legs of individual female mosquito specimens using the FavorPrep™ Mini Kit (Favorgen Biotech, Ping-Tung, Taiwan), according to the manufacturer’s protocol. All final DNA products were suspended in 50 μL elution buffer and stored at −20°C until PCR amplification.

We amplified an approximately 707-bp-long mitochondrial *COI* fragment using PCR with universal barcode primers COI_F (forward primer: 5′-GGA TTT GGA AAT TGA TTA GTT CCT T-3′) and COI_R (reverse primer: 5′-AAA AAT TTT AAT TCC AGT TGG AAC AGC-3′) recommended by Kumar et al. [48]. Each 25-μL PCR reaction mixture contained 4 μL of DNA template, 1× reaction buffer, 1.5 mM MgCl_2_, 0.2 mM dNTPs, 0.2 μM forward primer, 0.2 μM reverse primer, 5% dimethyl sulfoxide, 1.5 U Platinum Taq DNA polymerase (Invitrogen), and distilled water up to 25 μL. Negative controls were used in every PCR reaction by adding all reagents except genomic DNA. The thermal cycling conditions for PCR were set as follows: 5 min for initial denaturation at 95°C, followed by five cycles of denaturation for 40 s at 94°C, annealing for 60 s at 45°C, and extension for 1 min at 72°C; 35 cycles of denaturation for 40 s at 94°C, annealing for 60 s at 54°C, and extension for 1 min at 72°C, with final extension for 10 min at 72°C.

The quality of the PCR products was examined by electrophoresing the samples on 1.5% agarose gels and TBE buffer and staining with the Midori Green DNA stain (Nippon Gene, Tokyo, Japan). DNA was visualized using the ImageQuant LAS 500 imager (GE Healthcare Japan Corp., Tokyo, Japan) before nucleotide sequencing. The PCR products that showed clear DNA bands in a volume of 1 μL were sent to SolGent, Inc. (Daejeon, Korea) for nucleotide sequencing in both directions using the PCR primers.

### Sequence analyses

We manually edited the obtained trace files of the *COI* sequences, removed ambiguous bases, and created consensus sequences of each specimen based on forward and reverse sequences using the BioEdit software [49]. We excluded the samples with abnormalities in the nucleotide sequences, including ambiguities between sequences and double peaks, from genetic analyses to prevent problems from nuclear mitochondrial pseudogenes [50]. Multiple sequence alignments were performed using Clustal W [51] incorporated into MEGA X [52], with adjustment manually. Next, the consensus sequences, with detailed specimen field data, were uploaded to the Barcode of Life Data Systems (BOLD) database. The Refined Single Linkage (RESL) algorithm in BOLD was automatically used to assign the barcode sequences of mosquito species to Barcode Index Numbers (BINs) for assigning Operational Taxonomic Units (OTUs) (the proxy for species) [53]. Specimens assigned in the same BIN were presumed to belong to the same mosquito species.

For genetic distances, the intraspecific sequence divergence for each species was estimated using the nucleotide substitution model Kimura 2 Parameters (K2P) under MEGA X [50]. The distribution of the genetic distance to the nearest neighbor (NN) of each species was estimated based on K2P using the barcode gap analysis function in BOLD. Sequence polymorphic sites and number of haplotypes were estimated using DNA Sequences Polymorphism software version 6 [54]. The presence or absence of the “barcoding gap” of the mosquito species was evaluated using intraspecific values and the minimum interspecific distance (NN distance) [55].

The maximum likelihood (ML) tree—based on the general time-reversible model, with gamma-distribution rates plus invariant sites (GTR + G + I)—was constructed using MEGA X with bootstrapping (1,000 replicates) to visualize the genetic relationships between mosquito species belonging to the Anophelinae and Culicinae subfamilies, as described by Harbach [1]. All phylogenetic trees were edited and graphically prepared using FigTree v.1.4.3. (http://tree.bio.ed.ac.uk/software/Figtree/).

The assemble species by automatic partitioning (ASAP) method based on the simple distance (*p*-distances) [56] and the BIN-RESL algorithm method were used for molecular species delimitation. The TaxonDNA software [57] was used to diagnose identification success, ambiguous sequences, and misidentified sequences in the *COI* barcode dataset based on three query identification analyses to investigate the frequency of successful identification. These included the “Best Match” (considered from sequences with the smallest genetic distance to query all conspecifics [the closest match]), “Best Close Match” (considered from sequences with the smallest genetic distance to query all conspecifics and under the threshold value of 95% for all intraspecific genetic distances), and “All Species Barcodes” (considered from all conspecific sequences topping the list of the best matches and within the same threshold value as the best close match) methods [57]. In this study, the thresholds for “Best Close Match” and “All Species Barcodes” were set at 1% based on the standard cutoff value fixed by the BOLD database [58]. After that “Identification Engine” in BOLD was used to test the identification efficiency of the *COI* sequences obtained from the present study. This tool can help assess the identification efficiency of the *COI* sequences once compare with available *COI* sequences reported in the public database.

## Results

A total of 73 identified species from 14 genera were collected from several provinces in Thailand: *Aedes* (7 species), *Aedeomyia* (1 species), *Anopheles* (30 species), *Armigeres* (4 species), *Collessius* (1 species), *Coquillettidia* (2 species), *Culex* (15 species), *Lutzia* (3 species), *Mansonia* (5 species), *Mimomyia* (1 species), *Ochlerotatus* (1 species), *Rhinoskusea* (1 species), *Toxorhynchites* (1 species), and *Uranotaenia* (1 species) (S1 Table). A total of 310 mosquito samples, representing 73 mosquito species, were amplified using *COI* primers. These *COI* barcode sequences (not revealing insertions, deletions, stop codons, and pseudogenes) were submitted to the GenBank and BOLD databases.

### DNA sequence analyses

All *COI* sequences of the mosquito specimens were found to be adenosine- and thymine-rich (AT-rich) (an average of 68.4%), with an average nucleotide composition of thymine (T) = 38.5%, adenine (A) = 29.9%, cytosine (C) = 15.9%, and guanine (G) = 15.7%. DNA polymorphism analyses of the 310 *COI* sequences showed 417 monomorphic variable, 277 polymorphic variable, 271 parsimony informative, and 6 singleton variable sites. Haplotype analyses revealed 225 haplotypes from the 310 *COI* sequences (Table 1).

**Table 1:** List of mosquito species collected in this study, their locations, GenBank accession numbers of their cytochrome c oxidase subunit I (*COI*) sequences, number of haplotypes, mean intraspecific distances, polymorphic sites, and haplotype diversity.

Mosquito species	*n*	Locations	Accession numbers	h	% Mean distance (min–max)	Polymorphic sites	Haplotype diversity (± SD)
*Aedeomyia catasticta*	1	Kanchanaburi	OL743097	4	0.6 (0.3–0.7)	8	1.0 ± 0.2
	1	Trat	OL743099				
	1	Nakhon Ratchasima	OL743100				
	1	Surat Thani	OL743101				
*Aedes aegypti*	1	Kanchanaburi	OL742798	3	1.7 (0–2.8)	20	0.8 ± 0.2
	1	Nakhon Pathom	OL742799				
	1	Chanthaburi	OL742800				
	1	Surin	OL742801				
*Ae*. *albopictus*	1	Chiang Mai	OL742802	3	0.2 (0–0.4)	3	0.7 ± 0.2
	1	Kanchanaburi	OL742803				
	1	Ubon Ratchathani	OL742804				
	1	Chanthaburi	OL742805				
	1	Phang Nga	OL742806				
*Ae*. *desmotes*	2	Tak	OL742807-08	2	0.1 (0.1–0.1)	1	1.0 ± 0.5
*Ae*. *lineatopennis*	1	Tak	OL742809	4	1.0 (0.1–1.3)	12	1.0 ± 0.2
	1	Kanchanaburi	OL742810				
	1	Nakhon Ratchasima	OL742811				
	1	Narathiwat	OL742812				
*Ae*. *poicilius*	2	Tak	OL742813-14	1	0.0 (0.0)	0	0.0 ± 0.0
*Ae*. *vexans*	1	Chiang Mai	OL742815	7	0.8 (0.1–1.4)	15	1.0 ± 0.1
	1	Nan	OL742816				
	1	Kanchanaburi	OL742817				
	1	Ratchaburi	OL742818				
	1	Nakhon Pathom	OL742819				
	1	Nakhon Ratchasima	OL742820				
	1	Narathiwat	OL742821				
*Ae*. *vittatus*	3	Chiang Mai	OL742826-28	3	0.5 (0.0–1.1)	8	0.7 ± 0.2
	2	Kanchanaburi	OL742829-30				
*Anopheles aconitus*	1	Ratchaburi	OL742831	5	0.9 (0.1–2.0)	16	1.0 ± 0.1
	4	Trat	OL742832-35				
*An*. *annularis*	4	Ratchaburi	OL742836-37	4	2.6 (0–4.3)	32	0.8 ± 0.2
			OL744383-84				
	2	Narathiwat	OL742838				
			OL744382				
*An*. *baimaii*	2	Kanchanaburi	OL742839-40	2	0.7 (0.7–0.7)	5	1.0 ± 0.5
*An*. *culicifacies*	2	Tak	OL742841-42	1	0.0 (0.0)	0	0.0 ± 0.0
*An*. *dirus*	1	Chiang Mai	OL742843	6	1.2 (0.1–1.9)	16	1.0 ± 0.1
	3	Kanchanaburi	OL742844-46				
	2	Chachoengsao	OL742847-48				
*An*. *dissidens*	1	Trat	OL742849	2	0.3 (0.3–0.3)	2	1.0 ± 0.5
	1	Narathiwat	OL742850				
*An*. *dravidicus*	6	Kanchanaburi	OL742851-56	3	0.1 (0–0.3)	2	0.6 ± 0.2
*An*. *epiroticus*	1	Trat	OL742857	4	1.6 (0–2.6)	20	0.9 ± 0.2
	1	Phang Nga	OL742858				
	1	Suratthani	OL742859				
	1	NakhonSi Thammarat	OL742860				
	1	Krabi	OL742861				
*An*. *harrisoni*	3	Kanchanaburi	OL742862-64	3	0.6 (0.3–0.9)	6	1.0 ± 0.3
*An*. *jamesii*	1	Trat	OL742865	4	1.0 (0.1–1.7)	13	1.0 ± 0.2
	2	Phang Nga	OL742866-67				
	1	Krabi	OL742868				
*An*. *maculatus*	3	Trat	OL742869-71	4	0.3 (0–0.4)	5	0.9 ± 0.2
	2	Tak	OL742872-73				
*An*. *minimus*	2	Kanchanaburi	OL742874-75	3	0.6 (0–1.0)	7	0.8 ± 0.2
	1	Chachoengsao	OL742876				
	2	Phang Nga	OL742877-78				
*An*. *nemophilous*	1	Ratchaburi	OL742879	1	N/C	N/C	N/C
*An*. *nigerrimus*	1	Narathiwat	OL742880	1	N/C	N/C	N/C
*An*. *nitidus*	3	Narathiwat	OL742881-83	3	0.2 (0.1–0.3)	2	1.0 ± 0.3
*An*. *nivipes*	1	Chachoengsao	OL742884	2	0.7 (0.7–0.7)	5	1.0 ± 0.5
	1	Ubon Ratchathani	OL742885				
*An*. *paraliae*	4	Ratchaburi	OL742886-89	3	0.3 (0–1.0)	7	0.6 ± 0.2
	2	Samut Songkhram	OL742890-91				
*An*. *peditaeniatus*	1	Ratchaburi	OL742892	1	N/C	N/C	N/C
*An*. *philippinensis*	2	Ubon Ratchathani	OL742893-94	2	0.3 (0–0.4)	3	0.7 ± 0.2
	1	Narathiwat	OL742895				
	1	Trat	OL742896				
*An*. *pseudojamesi*	1	Chiang Mai	OL742897	6	1.4 (0.1–2.0)	21	1.0 ± 0.1
	1	Chachoengsao	OL742898				
	2	Trat	OL742899-900				
	2	Narathiwat	OL742901-02				
*An*. *pseudowillmori*	2	Tak	OL742903-04	2	1.0 (1.0–1.0)	7	1.0 ± 0.5
*An*. *pursati*	3	Samut Songkhram	OL742905-07	3	0.5 (0.4–0.6)	5	1.0 ± 0.3
*An*. *saeungae*	1	Ubon Ratchathani	OL742908	2	0.7 (0.7–0.7)	5	1.0 ± 0.5
	1	Nakhon Si Thammarat	OL742910				
*An*. *sawadwongporni*	3	Tak	OL742914-16	4	0.2 (0–0.3)	3	0.9 ± 0.1
	3	Ratchaburi	OL742917-19				
*An*. *sinensis*	1	Ratchaburi	OL742920	2	0.3 (0.3–0.3)	2	1.0 ± 0.5
	1	Chaiyaphum	OL742921				
*An*. *subpictus*	1	Ratchaburi	OL742922	3	1.6 (0–2.9)	21	0.8 ± 0.2
	1	Nakhon Pathom	OL742923				
	1	Chachoengsao	OL742924				
	1	Chaiyaphum	OL742925				
*An*. *tessellatus*	4	Nan	OL742926-29	5	2.5 (0.3–5.7)	42	1.0 ± 0.1
	1	Phang Nga	OL742930				
*An*. *vagus*	2	Ratchaburi	OL742931-32	3	1.1 (1.0–1.1)	11	1.0 ± 0.3
	1	NakhonPathom	OL742933				
*An*. *varuna*	1	Kanchanaburi	OL742934	2	1.0 (1.0–1.0)	7	1.0 ± 0.5
	1	Ratchaburi	OL742935				
*An*. *wejchoochotei*	2	Kanchanaburi	OL742936-37	4	0.3 (0–0.7)	6	0.9 ± 0.1
	2	Samut Songkhram	OL742938-39				
	2	Chachoengsao	OL742940-41				
	2	Chanthaburi	OL742942-43				
*Armigeres durhami*	6	Chiang Mai	OL742949-54	1	0.0 (0.0)	0	0.0 ± 0.0
*Ar*. *flavus*	2	Ratchaburi	OL742955-56	1	0.0 (0.0)	0	0.0 ± 0.0
*Ar*. *malayi*	1	Ratchaburi	OL742957	1	N/C	N/C	N/C
*Ar*. *subalbatus*	1	Chiang Mai	OL742944	2	0.1 (0–0.1)	1	0.4 ± 0.2
	1	Kanchanaburi	OL742945				
	1	Ubon Ratchathani	OL742946				
	1	Chanthaburi	OL742947				
	1	Phang Nga	OL742948				
*Collessius macfarlanei*	4	Ubon Ratchathani	OL743102-05	1	0.0 (0.0)	0	0.0 ± 0.0
*Coquillettidia crassipes*	1	Chiang Mai	OL742958	4	1.3 (0.7–1.7)	18	1.0 ± 0.2
	1	Trat	OL742959				
	1	Ubon Ratchathani	OL742960				
	1	Phang Nga	OL742961				
*Cq*. *ochracea*	1	Chanthaburi	OL742962	2	0.5 (0–1.6)	11	0.3 ± 0.2
	5	Narathiwat	OL742963-67				
*Culex bicornutus*	2	Kanchanaburi	OL742968-69	4	0.2 (0–0.4)	3	0.9 ± 0.1
	6	Ratchaburi	OL742970-75				
*Cx*. *bitaeniorhynchus*	2	Mae Hong Son	OL742976-77	3	0.1 (0–0.3)	2	0.8 ± 0.2
	2	Ratchaburi	OL742978-79				
*Cx*. *brevipalpis*	1	Kanchanaburi	OL742980	3	0.4 (0–0.9)	6	0.8 ± 0.2
	2	Ratchaburi	OL742981-82				
	1	Ubon Ratchathani	OL742983				
*Cx*. *epidesmus*	3	Tak	OL742984-86	3	0.8 (0.6–1.0)	8	1.0 ± 0.3
*Cx*. *fuscocephala*	1	Mae Hong Son	OL742987	1	0.0 (0.0)	0	0.0 ± 0.0
	1	Chiang Mai	OL742988				
	1	Ubon Ratchathani	OL742989				
	2	Phang Nga	OL742990-91				
*Cx*. *gelidus*	1	Chiang Mai	OL742992	3	0.6 (0–1.9)	13	0.6 ± 0.2
	1	Ratchaburi	OL742993				
	1	Samut Songkhram	OL742994				
	1	Chachoengsao	OL742995				
	1	Ubon Ratchathani	OL742996				
	1	Phang Nga	OL742997				
*Cx*. *infantulus*	4	Kanchanaburi	OL742998-3001	6	0.6 (0–0.9)	11	0.9 ± 0.1
	4	Ratchaburi	OL743002-05				
*Cx*. *murrelli*	1	Chiang Mai	OL743006	5	0.3 (0–0.4)	5	0.9 ± 0.1
	1	Ratchaburi	OL743007				
	1	Chanthaburi	OL743008				
	1	Trat	OL743009				
	2	Surat Thani	OL743010-11				
*Cx*. *nigropunctatus*	1	Chiang Mai	OL743012	7	0.9 (0.1–1.6)	17	1.0 ± 0.1
	1	Kanchanaburi	OL743013				
	1	Ratchaburi	OL743014				
	1	Phetchaburi	OL743015				
	1	Trat	OL743016				
	1	Nakhon Ratchasima	OL743017				
	1	Ubon Ratchathani	OL743018				
*Cx*. *pallidothorax*	3	Tak	OL743019-21	5	0.6 (0–0.9)	9	0.9 ± 0.1
	3	Kanchanaburi	OL743022-24				
	1	Ubon Ratchathani	OL743025				
*Cx*. *pseudovishnui*	2	Narathiwat	OL743028-29	1	0.00 (0.00)	0	0.0 ± 0.0
*Cx*. *quinquefasciatus*	1	Chiang Mai	OL743030	2	0.1 (0–0.1)	1	0.4 ± 0.2
	1	Kanchanaburi	OL743031				
	1	Ratchaburi	OL743032				
	1	Nakhon Pathom	OL743033				
	2	Trat	OL743034-35				
	1	Ubon Ratchathani	OL743036				
	1	Nakhon Si Thammarat	OL743037				
*Cx*. *sitiens*	1	Samut Songkhram	OL743038	3	0.4 (0–0.7)	5	0.7 ± 0.2
	1	Trat	OL743039				
	1	Phang Nga	OL743040				
	2	Surat Thani	OL743041-42				
	2	Krabi	OL743043-44				
*Cx*. *tritaeniorhynchus*	2	Chiang Mai	OL743045-46	6	0.9 (0.1–1.9)	18	1.0 ± 0.1
	1	Ratchaburi	OL743047				
	1	Samut Songkhram	OL743048				
	1	Chanthaburi	OL743049				
	1	Ubon Ratchathani	OL743050				
*Cx*. *vishnui*	1	Chiang Mai	OL743051	6	0.6 (0–1.1)	9	1.0 ± 0.1
	2	Kanchanaburi	OL743052-53				
	4	Ubon Ratchathani	OL743054-57				
*Lutzia vorax*	1	Ubon Ratchathani	OL743058	2	1.3 (1.3–1.3)	9	1.0 ± 0.5
	1	Kanchanaburi	OL743059				
*Lt*. *fuscana*	3	Ratchaburi	OL743060-62	5	0.6 (0–0.9)	10	0.9 ± 0.1
	3	Chachoengsao	OL743063-65				
*Lt*. *chiangmaiensis*	1	Ubon Ratchathani	OL743066	3	0.1 (0–0.3)	2	0.7 ± 0.2
	3	Ratchaburi	OL743067-69				
	1	Kanchanaburi	OL743070				
	1	Chachoengsao	OL743071				
*Mansonia annulifera*	1	Chiang Mai	OL743072	6	1.5 (0.6–2.2)	23	1.0 ± 0.1
	1	Ratchaburi	OL743073				
	1	Samut Songkhram	OL743074				
	1	Trat	OL743075				
	1	Nakhon Ratchasima	OL743076				
	1	Narathiwat	OL743077				
*Ma*. *bonneae*	5	Narathiwat	OL743078-82	5	0.8 (0.1–1.1)	11	1.0 ± 0.1
*Ma*. *dives*	2	Tak	OL743083-84	2	1.6 (1.6–1.6)	11	1.0 ± 0.5
*Ma*. *indiana*	2	Kanchanaburi	OL743087-88	2	0.3 (0–0.4)	3	0.7 ± 0.3
	1	Ratchaburi	OL743089				
*Ma*. *uniformis*	1	Chiang Mai	OL743090	4	0.3 (0–0.9)	6	0.8 ± 0.2
	1	Ratchaburi	OL743091				
	1	Samut Songkhram	OL743092				
	1	Trat	OL743093				
	1	Ubon Ratchathani	OL743094				
	1	Surat Thani	OL743095				
*Mimomyia aurea*	3	Narathiwat	OL743106-08	2	0.1 (0–0.1)	1	0.7 ± 0.3
*Ochlerotatus vigilax*	4	Phang Nga	OL742822-25	3	0.3 (0–0.6)	4	0.8 ± 0.2
*Rhinoskusea longirostris*	2	Ratchaburi	OL743109-10	2	0.7 (0.7–0.7)	5	1.0 ± 0.5
*Toxorhynchites splendens*	2	Surat Thani	OL743111-12	1	0.0 (0.0)	0	0.0 ± 0.0
*Uranotaenia obscura*	2	Ratchaburi	OL743113-14	1	0.0 (0.0)	0	0.0 ± 0.0

n = total number of *COI* sequences; h = number of haplotypes; min = minimum; max = maximum; SD = standard deviation; N/C = not calculated (species represented by only one specimen). Detailed collection- and geographic coordinate–related data are indicated in S1 Table.

### BIN analysis

A total of 310 barcode sequences were assigned to the existing BINs in BOLD based on OTUs by the RESL algorithm. BIN analysis revealed that our *COI* dataset contained 75 BINs, representing 73 mosquito species (Table 2). Sixty-six species perfectly clustered into a single BIN. Four species were split into two BINs, namely: *An*. *annularis* (BOLD: AAR3272 and AEG7105), *An*. *dirus* (BOLD: AAC7100 and ABZ2357), *An*. *subpictus* (BOLD: AAA4215 and ABY5601), and *An*. *tessellatus* (BOLD: AAT9116 and ACW0305). Two species pairs including *An*. *baimaii* and *An*. *dirus* (BOLD: ABZ2357), and *Lt*. *fuscana* and *Lt*. *chiangmaiensis* (BOLD: AAG3834) shared the same BINs.

**Table 2:** Barcode index number details and genetic distance to the nearest species (minimum interspecific distance) based on barcode gap analysis in the barcode of life data systems.

Mosquito species	Barcode Index Number (BIN) details			The nearest species	Distance to the nearest neighbour
	Barcode index numbers ID	Total members	Count in project		
*Ad*. *Catasticta*	ACV6316	5	4	*Lt*. *chiangmaiensis*	10.6
*Ae*. *Aegypti*	AAA4210	2193	4	*Ae*. *vexans*	9.2
*Ae*. *Albopictus*	AAA5870	3776	5	*Ae*. *lineatopennis*	10.2
*Ae*. *Desmotes*	ADK1864	32	2	*Ae*. *vittatus*	9.5
*Ae*. *Lineatopennis*	ACB9609	12	4	*Ae*. *vittatus*	7.6
*Ae*. *Poicilius*	AEK4068	3	2	*Cx*. *sitiens*	10.4
*Ae*. *Vexans*	AAA7067	4873	7	*Ae*. *vittatus*	8.1
*Ae*. *Vittatus*	AAV4160	120	5	*Ae*. *lineatopennis*	7.6
*An*. *Aconitus*	AAB2519	42	5	*An*. *minimus*	8.1
*An*. *Annularis*	AAR3272	91	3	*An*. *culicifacies*	7.6
	AEG7105	8	3		
*An*. *Baimaii*	ABZ2357	24	2	*An*. *dirus*	0.3
*An*. *Culicifacies*	AAF0681	40	2	*An*. *minimus*	7.1
*An*. *Dirus*	AAC7100	11	3	*An*. *baimaii*	0.3
	ABZ2357	24	3		
*An*. *Dissidens*	AAA5684	142	2	*An*. *saeungae*	3.3
*An*. *Dravidicus*	ABY9593	14	6	*An*. *maculatus*	6.3
*An*. *Epiroticus*	AAA4214	169	5	*An*. *culicifacies*	9.5
*An*. *Harrisoni*	ACF2761	49	3	*An*. *minimus*	3.0
*An*. *Jamesii*	AAD2562	43	4	*An*. *maculatus*	8.8
*An*. *Maculatus*	AAC3408	39	5	*An*. *dravidicus*	6.3
*An*. *Minimus*	AAB5964	39	5	*An*. *harrisoni*	3.0
*An*. *Nemophilous*	AEA5944	23	1	*An*. *dirus*	5.6
*An*. *Nigerrimus*	AAI4557	22	1	*An*. *pursati*	5.8
*An*. *Nitidus*	AAA5749	53	3	*An*. *pursati*	4.0
*An*. *Nivipes*	AAD9018	37	2	*An*. *philippinensis*	7.3
*An*. *Paraliae*	AAA5748	113	6	*An*. *peditaeniatus*	4.1
*An*. *Peditaeniatus*	AAD3070	163	1	*An*. *paraliae*	4.1
*An*. *Philippinensis*	ADS6721	5	4	*An*.*nivipes*	7.3
*An*. *Pseudojamesi*	AEQ7311	6	6	*An*. *philippinensis*	8.5
*An*. *pseudowillmori*	AAJ2796	18	2	*An*. *dirus*	9.3
*An*. *Pursati*	ACQ5333	19	3	*An*. *nitidus*	4.0
*An*. *Saeungae*	ACF3961	11	2	*An*. *wejchoochotei*	1.9
*An*. *sawadwongporni*	AAC3407	44	6	*An*. *maculatus*	6.8
*An*. *Sinensis*	AAA5339	726	2	*An*. *paraliae*	4.3
*An*. *Subpictus*	AAA4215	20	3	*An*. *paraliae*	9.5
	ABY5601	10	1		
*An*. *Tessellatus*	AAT9116	46	4	*An*. *saeungae*	7.8
	ACW0305	5	1		
*An*. *Vagus*	AAE4158	63	3	*An*. *paraliae*	10.1
*An*. *Varuna*	AAD5962	19	2	*An*. *culicifacies*	8.2
*An*. *wejchoochotei*	AAA5682	84	8	*An*. *saeungae*	1.9
*Ar*. *Durhami*	ADU2633	13	6	*Ar*. *subalbatus*	9.1
*Ar*. *Flavus*	ACP1995	24	2	*Ar*. *malayi*	9.9
*Ar*. *Malayi*	ADT8626	2	1	*Ar*. *subalbatus*	9.4
*Ar*. *Subalbatus*	AAC8113	428	5	*Ae*. *lineatopennis*	8.2
*Co*. *macfarlanei*	AEP6081	4	4	*Ae*. *vigilax*	9.5
*Cq*. *Crassipes*	AAU6779	15	4	*Cq*. *ochracea*	11.7
*Cq*. *Ochracea*	ADJ5998	9	6	*Co*. *macfarlanei*	10.3
*Cx*. *Bicornutus*	ACC8728	25	8	*Cx*. *epidesmus*	8.3
*Cx*. *bitaeniorhynchus*	AAJ7281	100	4	*Cx*. *epidesmus*	4.6
*Cx*. *Brevipalpis*	ACC8753	8	4	*Cq*. *ochracea*	10.6
*Cx*. *Epidesmus*	AAZ7107	10	3	*Cx*. *bitaeniorhynchus*	4.6
*Cx*. *Fuscocephala*	AAJ7295	51	5	*Cx*. *epidesmus*	6.6
*Cx*. *Gelidus*	AAC6669	157	6	*Cx*. *sitiens*	5.3
*Cx*. *Infantulus*	AAZ3192	14	8	*Cx*. *gelidus*	6.6
*Cx*. *Murrelli*	ACC8692	11	6	*Cx*. *bitaeniorhynchus*	6.3
*Cx*. *nigropunctatus*	AAR4910	41	7	*Cx*. *pallidothorax*	4.1
*Cx*. *Pallidothorax*	AAZ3501	143	7	*Cx*. *nigropunctatus*	4.1
*Cx*. *Pseudovishnui*	ACM5555	14	2	*Cx*. *vishnui*	4.5
*Cx*. *quinquefasciatus*	AAA4751	6459	8	*Cx*. *pseudovishnui*	7.3
*Cx*. *Sitiens*	AAB7378	189	7	*Cx*. *gelidus*	5.3
*Cx*. *tritaeniorhynchus*	AAE3201	2716	6	*Cx*. *vishnui*	4.8
*Cx*. *Vishnui*	AAI9879	41	7	*Cx*. *pseudovishnui*	4.5
*Lt*. *vorax*	ACC8609	43	2	*Lt*. *fuscana*	4.5
*Lt*. *fuscana*	AAG3834	69	6	*Lt*. *chiangmaiensis*	1.6
*Lt*. *chiangmaiensis*	AAG3834	69	6	*Lt*. *fuscana*	1.6
*Ma*. *Annulifera*	AAF2325	27	6	*Ma*. *bonneae*	12.3
*Ma*. *Bonneae*	AAO3240	48	5	*Ma*. *dives*	4.8
*Ma*. *Dives*	ADS1936	43	2	*Ma*. *bonneae*	4.8
*Ma*. *Indiana*	AAC8223	24	3	*Ma*. *bonneae*	11.1
*Ma*. *Uniformis*	AAB7890	93	6	*Ma*. *bonneae*	9.7
*Mi*. *aurea*	AEP3699	3	3	*Lt*. *chiangmaiensis*	12.4
*Oc*. *vigilax*	AAC1707	250	4	*Ae*. *lineatopennis*	9.5
*Rh*. *Longirostris*	AEP6082	2	2	*Ae*. *lineatopennis*	9.0
*Tx*. *Splendens*	AAC7483	13	2	*Ae*. *poicilius*	12.9
*Ur*. *Obscura*	AEI3994	4	2	*Ae*. *poicilius*	12.0

In this study, four mosquito species were identified as new *COI* sequence records: *An*. *pseudojamesi*, *Collessius macfarlanei*, *Mimomyia aurea*, and *Rhinoskusea longirostris* and were assigned unique BINs: AEQ7311, AEP6081, AEP3699, and AEP6082, respectively.

### Sequence divergence

The average intraspecific genetic variation of the 73 mosquito species, based on the K2P distance method, was 0.6% (range = 0–2.6%). The highest average intraspecific divergence was observed in *An*. *annularis* at 2.6% (range = 0–4.3%), followed by *An*. *tessellatus* at 2.5% (range = 0.3–5.7%) (Table 1). While, average minimum interspecific genetic variation (the distance to the nearest neighbour) of the 73 mosquito species was 7% ranged from 0.3–12.9%. The greatest minimum interspecific divergence was found in *Tx*. *splendens* (closest to *Ae*. *poicilius*, 12.9%), followed by *Mi*. *aurea* (closest to *Lt*. *chiangmaiensis*, 12.4%) (Table 2). The lowest minimum interspecific divergence was found in *An*. *baimaii* (closest to *An*. *dirus*, 0.3%) and *An*. *dirus* (closest to *An*. *baimaii*, 0.3%), followed by *Lt*. *fuscana* (closest to *Lt*. *chiangmaiensis*, 1.6%) and *Lt*. *chiangmaiensis* (closest to *Lt*. *fuscana*, 1.6%).

### Barcoding gap analysis and species identification efficiency

**Fig 2:**
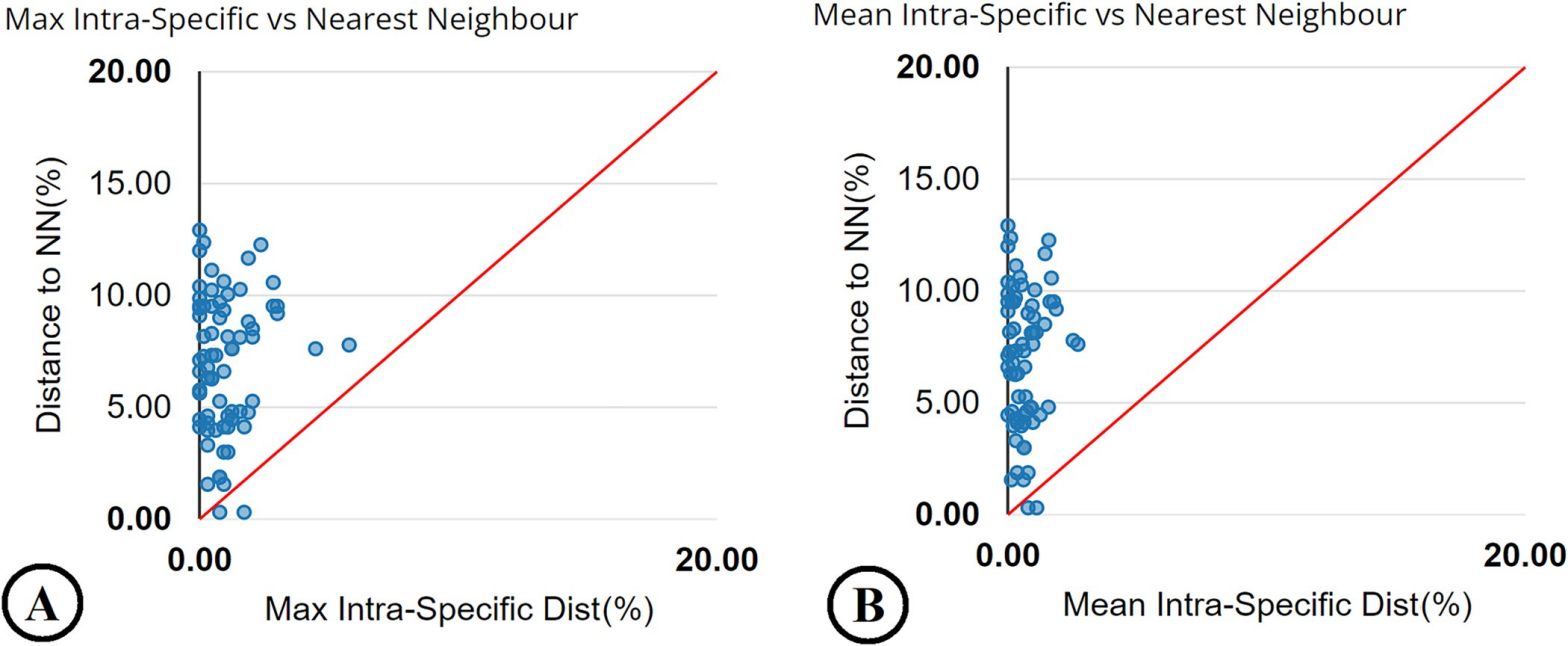
Scatter plots based on barcode gap analysis of 73 mosquito species. (A) Maximum intraspecific distances compared with minimum interspecific distances (distance to the nearest species), and (B) Mean intraspecific distances compared with minimum interspecific distances. Species dots above the 1:1 line show the presence of a “barcode gap,” whereas those on and below the 1:1 line show the absence of a “barcode gap”.

**Fig 3:**
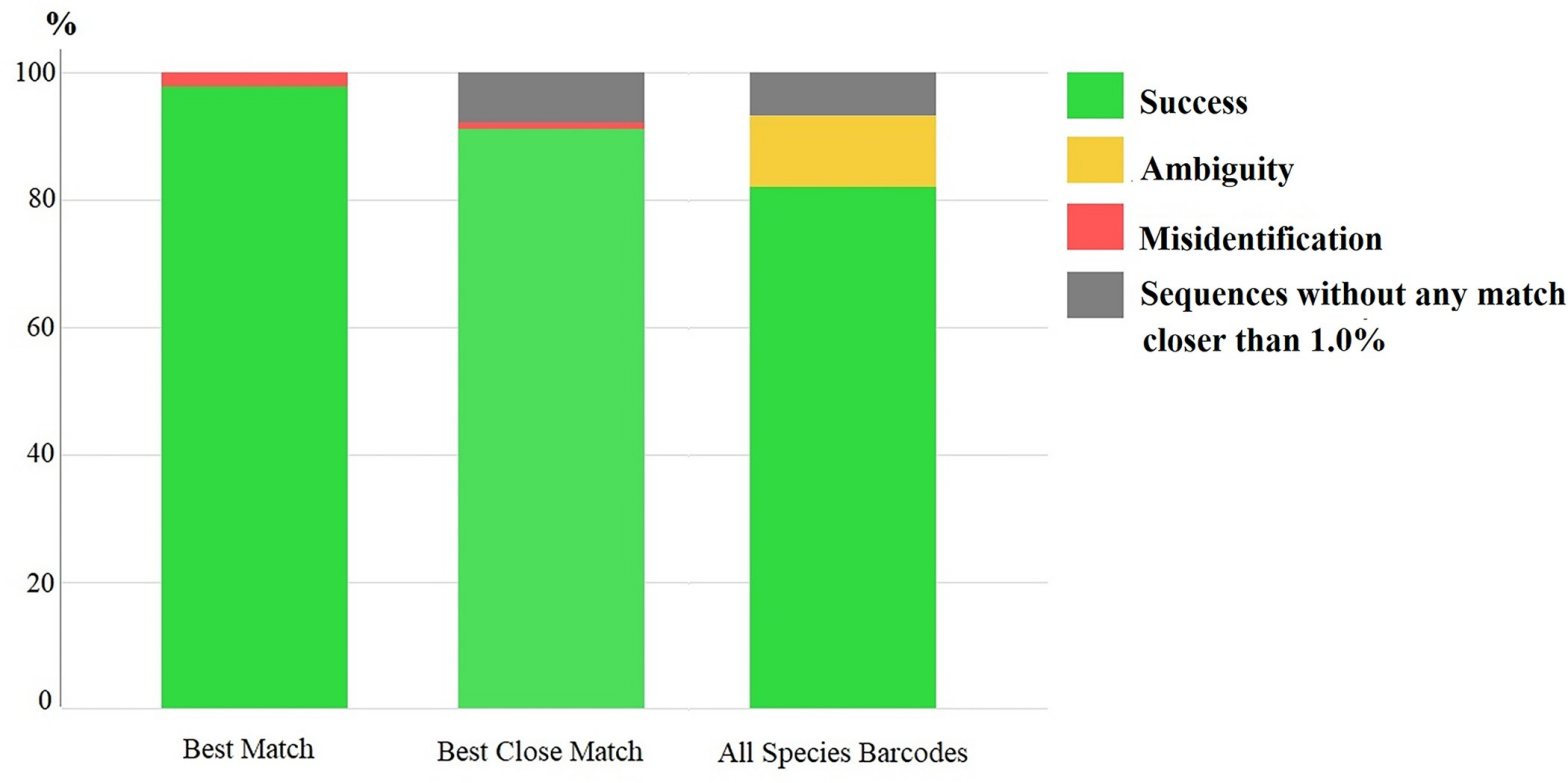
Results of specimen identification success based on the “Best Match,” “Best Close Match,” and “All Species Barcodes” methods. The threshold value for “Best Close Match” and “All Species Barcodes” was set at 1%.

To estimate the presence or absence of a “barcode gap,” intraspecific genetic distance was compared with the minimum interspecific genetic distance. Barcode gap analysis revealed that *An*. *baimaii* and *An*. *dirus* had overlap (absence of a barcode gap) (Fig 2). The identification success rates of our *COI* barcode sequences based on the “Best Match,” “Best Close Match,” and “All Species Barcodes” methods were 97.7%, 91.6%, and 81%, respectively; those of ambiguous identification were 0%, 0%, and 11.6%, respectively; and those of incorrect identification were 2.3%, 1%, and 0%, respectively (Fig 3). The percentages of sequences without any match closer than 1% in the “Best Close Match” and “All Species Barcodes” methods were the same (at 7.4%).

Comparisons of the *COI* sequences in this study with all available *COI* sequences reported in the public database were performed using the BOLD Identification Engine. Sixty-six species were correctly identified (based on top matches, Table 3). Whereas three mosquito species were ambiguous including *Ae*. *vittatus* (confused with *Ae*. *cogilli*), *An*. *dirus* (confused with *An*. *baimaii*) and *An*. *baimaii* (confused with *An*. *dirus*). In addition, high levels of genetic diversity based on the gap between maximum and minimum values in some mosquito species indicated that they are species complex such as *An*. *annularis* (93.3%–100%), *An*. *culicifacies* (94.8%–99.8%), *An*. *maculatus* (93%–100%), *An*. *subpictus* (91.1%–100%), and *An*. *tessellatus* (92.1%–99.8%).

**Table 3:** Results of BOLD identification engine.

Mosquito species in present study	*n*	Average match (%)	Min–Max (%)	Match species in Bold database	Match sequence number from queries to the BOLD ID engine	
*Ad*. *Catasticta*	4	98.3	97.0–99.5	*Ad*. *catasticta*		6
*Ae*. *Aegypti*	4	99.6	98.5–100	*Ae*. *aegypti*		100
*Ae*. *Albopictus*	5	99.9	99.8–100	*Ae*. *albopictus*		101
*Ae*. *Desmotes*	2	99.0	97.0–99.7	*Ae*. *desmotes*		13
*Ae*. *lineatopennis*	4	98.6	97.6–99.8	*Ae*. *lineatopennis*		10
*Ae*. *Poicilius*	2	99.6	99.1–100	*Ae*. *poicilius*		2
*Ae*. *Vexans*	7	97.8	96.7–100	*Ae*. *vexans*		100
*Ae*. *Vittatus*	5	98.4	97.8–99.8	*Ae*. *vittatus*		53
		98.4	97.6–99.1	*Ae*. *cogilli*		47
*An*. *Aconitus*	5	97.9	95.21–99.8	*An*. *aconitus*		73
*An*. *Annularis*	6	97.0	93.3–100	*An*. *annularis*		99
*An*. *Baimaii*	2	99.2	99.2–99.2	*An*. *baimaii*		2
		98.8	97.4–100	*An*. *dirus*		35
*An*. *Culicifacies*	2	97.8	94.8–99.8	*An*. *culicifacies*		48
*An*. *Dirus*	6	98.7	97.3–100	*An*. *dirus*		34
		99.3	98.9–99.7	*An*. *baimaii*		2
*An*. *Dissidens*	2	99.6	99.6–99.8	*An*. *dissidens*		58
*An*. *Dravidicus*	6	98.8	96.5–99.8	*An*. *dravidicus*		14
*An*. *Epiroticus*	5	97.9	96.8–100	*An*. *epiroticus*		34
*An*. *Harrisoni*	3	99.1	98.5–99.6	*An*. *harrisoni*		13
*An*. *Jamesii*	4	99.0	96.3–99.8	*An*. *jamesii*		22
*An*. *Maculatus*	5	97.6	93.0–100	*An*. *maculatus*		70
*An*. *Minimus*	5	98.2	96.5–100	*An*. *minimus*		81
*An*. *nemophilous*	1	97.7	97.4–98.1	*An*. *introlatus*		21
*An*. *Nigerrimus*	1	98.6	95.3–99.6	*An*. *nigerrimus*		23
*An*. *Nitidus*	3	99.0	97.6–100	*An*. *nitidus*		46
*An*. *Nivipes*	2	98.9	98.5–99.6	*An*. *nivipes*		36
*An*. *Paraliae*	6	98.4	97.4–99.8	*An*. *lesteri*		99
*An*. *peditaeniatus*	1	99.6	99.3–100	*An*. *peditaeniatus*		100
*An*. *philippinensis*	4	98.0	95.2–99.9	*An*. *philippinensis*		5
*An*. *pseudowillmori*	2	99.3	98.5–100	*An*. *pseudowillmori*		16
*An*. *Pursati*	3	98.6	98.0–99.4	*An*. *pursati*		18
*An*. *Saeungae*	2	98.8	97.6–99.7	*An*. *saeungae*		24
*An*. *sawadwongporni*	6	99.4	98.7–99.8	*An*. *sawadwongporni*		22
*An*. *Sinensis*	2	98.8	98.7–99.5	*An*. *sinensis*		86
*An*. *Subpictus*	4	96.1	91.1–100	*An*. *subpictus*		19
*An*. *Tessellatus*	5	96.4	92.1–99.8	*An*. *tessellatus*		96
*An*. *Vagus*	3	98.3	95.6–100	*An*. *vagus*		48
*An*. *Varuna*	2	99.2	98.1–99.8	*An*. *varuna*		17
*An*. *wejchoochotei*	8	99.5	98.5–100	*An*. *wejchoochotei*		46
*Ar*. *Durhami*	6	99.7	99.2–100	*Ar*. *durhami*		12
*Ar*. *Flavus*	2	98.8	98.1–99.8	*Ar*. *flavus*		5
*Ar*. *Malayi*	1	99.6	99.6–99.6	*Ar*. *malayi*		1
*Ar*. *Subalbatus*	5	99.9	99.8–100	*Ar*. *subalbatus*		100
*Cq*. *Crassipes*	4	98.4	96.4–99.6	*Cq*. *crassipes*		16
*Cq*. *Ochracea*	6	99.4	98.5–99.4	*Cq*. *ochracea*		8
*Cx*. *Bicornutus*	8	99.3	98.25–100	*Cx*. *bicornutus*		13
*Cx*. *bitaeniorhynchus*	4	98.9	97.4–100	*Cx*. *bitaeniorhynchus*		73
*Cx*. *Brevipalpis*	4	97.0	94.4–100	*Cx*. *brevipalpis*		23
*Cx*. *Epidesmus*	3	99.2	98.5–99.8	*Cx*. *epidesmus*		8
*Cx*. *fuscocephala*	5	99.5	98.2–100	*Cx*. *fuscocephala*		48
*Cx*. *Gelidus*	6	99.1	98.1–100	*Cx*. *gelidus*		100
*Cx*. *Infantulus*	8	98.0	96.1–99.8	*Cx*. *infantulus*		28
*Cx*. *Murrelli*	6	98.9	96.1–99.8	*Cx*. *murrelli*		9
*Cx*. *nigropunctatus*	7	98.4	96.5–99.7	*Cx*. *nigropunctatus*		32
*Cx*. *pallidothorax*	7	98.9	98.3–99.5	*Cx*. *pallidothorax*		99
*Cx*. *pseudovishnui*	2	96.9	95.2–99.8	*Cx*. *pseudovishnui*		50
*Cx*. *quinquefasciatus*	8	99.9	99.8–100	*Cx*. *quinquefasciatus*		35
*Cx*. *Sitiens*	7	99.5	98.7–100	*Cx*. *sitiens*		98
*Cx*. *tritaeniorhynchus*	6	99.6	99.2–100	*Cx*. *tritaeniorhynchus*		100
*Cx*. *Vishnui*	7	99.2	95.2–100	*Cx*. *vishnui*		38
*Lt*. *vorax*	2	98.8	97.8–99.8	*Lt*. *vorax*		36
*Lt*. *fuscana*	6	99.0	96.9–100	*Lt*. *fuscana*		34
*Lt*. *chiangmaiensis*	6	99.4	98.3–100	*Lt*. *chiangmaiensis*		15
*Ma*. *Annulifera*	6	98.1	96.7–99.2	*Ma*. *annulifera*		26
*Ma*. *Bonneae*	5	98.9	98.3–99.8	*Ma*. *bonneae*		21
*Ma*. *Dives*	2	97.6	96.6–99.5	*Ma*. *dives*		16
*Ma*. *Indiana*	3	99.2	98.7–100	*Ma*. *indiana*		23
*Ma*. *Uniformis*	6	98.6	95.7–99.8	*Ma*. *uniformis*		80
*Oc*. *vigilax*	4	99.1	98.7–99.8	*Oc*. *vigilax*		100
*Tx*. *Splendens*	2	98.2	97.1–100	*Tx*. *splendens*		32
*Ur*. *Obscura*	2	99.4	98.9–100	*Ur*. *obscura*		3

Four species including *An*. *pseudojamesi*, *Co*. *macfarlanei*, *Mi*. *aurea*, and *Rh*. *longirostris* as new records are not included in the table.

### Phylogenetic analysis and species delimitations

**Fig 4:**
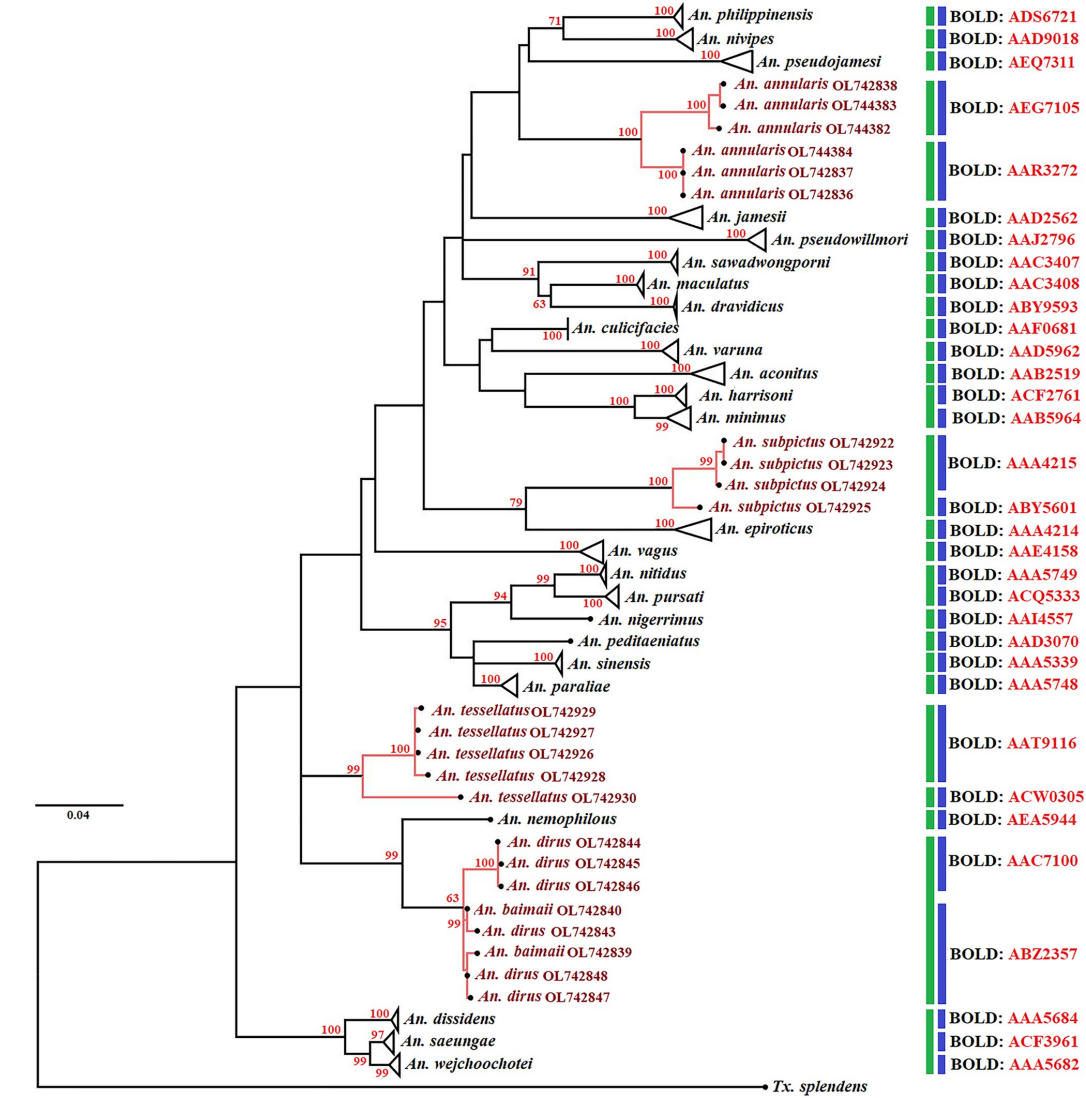
Maximum likelihood (ML) tree based on 112 cytochrome *c* oxidase subunit I (*COI*) sequences representing 30 mosquito species in the subfamily Anophelinae. Bootstrap values (1000 replicates) are shown near each branch (numbers in red). Vertical bars indicate species delimited using the assemble species by automatic partitioning (ASAP) (green bars) and the BIN-refined single linkage analysis (RESL) algorithm (blue bars) methods. *Toxorhynchites splendens* (OL743111) was used as an outgroup to root the tree. The pink branches showed subgrouping in the same mosquito species.

Maximum likelihood phylogenetic analysis in Fig 4 reveals the genetic relationships between 112 *COI* sequences, representing 30 morphologically identified species in the subfamily Anophelinae and *Tx*. *splendens* (OL743111) as an outgroup. Phylogenetic analyses of *Anopheles COI* sequences demonstrated a clear separation between almost all species, with high bootstrap support values (97%–99%), except for those between *An*. *dirus* and *An*. *baimaii*. Results from species delimitation of *Anopheles* mosquitoes based on the BIN-RESL algorithm were consistent with those of ML phylogenetic analysis (Fig 4). *Anopheles baimaii* and *An*. *dirus* are clustered together. Three species: *An*. *annularis*, *An*. *tessellatus*, and *An*. *subpictus* were split into two subclusters based on BIN assignment. By contrast, ASAP based on the simple distance failed to separate many *Anopheles* species pairs, including *An*. *minimus* and *An*. *harrisoni*, *An*. *nitidus* and *An*. *pursati*, and *An*. *dissidens* + *An*. *wejchoochotei* + *An*. *saeungae* (Fig 4).

**Fig 5:**
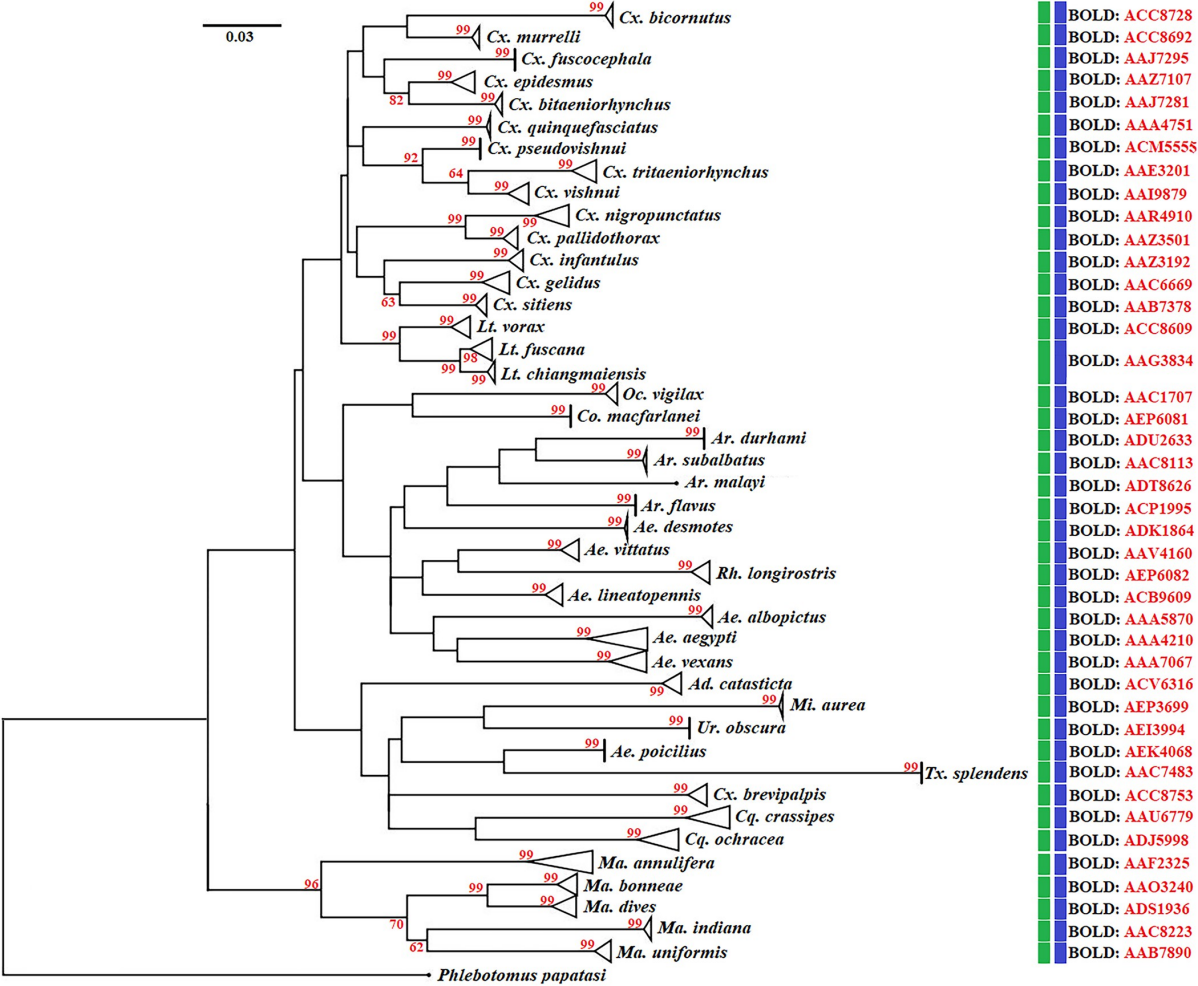
Maximum likelihood (ML) tree based on 198 cytochrome *c* oxidase subunit I (*COI*) sequences representing 43 mosquito species in the subfamily Culicinae. Bootstrap values (1000 replicates) are shown near each branch (numbers in red). Vertical bars indicate species delimited using the assemble species by automatic partitioning (ASAP) (green bars) and the BIN-refined single linkage analysis (RESL) algorithm (blue bars) methods. *Phlebotomus papatasi* (MN086383).

The phylogenetic tree of the mosquito species in the subfamily Culicinae based on 198 *COI* sequences representing 43 morphologically identified species is presented in Fig 5. All Culicinae mosquitoes showed a clear separation between species, with high bootstrap support values (98–100%), whereas *Phlebotomus papatasi* (MN086383), an outgroup species, was distinct from other mosquito species. The BIN-RESL algorithm and ASAP delimited mosquito species of the subfamily Culicinae into 42 taxa, as *Lt*. *fuscana* and *Lt*. *chiangmaiensis* were not separated, which is not consistent with the results of the ML phylogenetic analysis (Fig 5).

**Fig 6:**
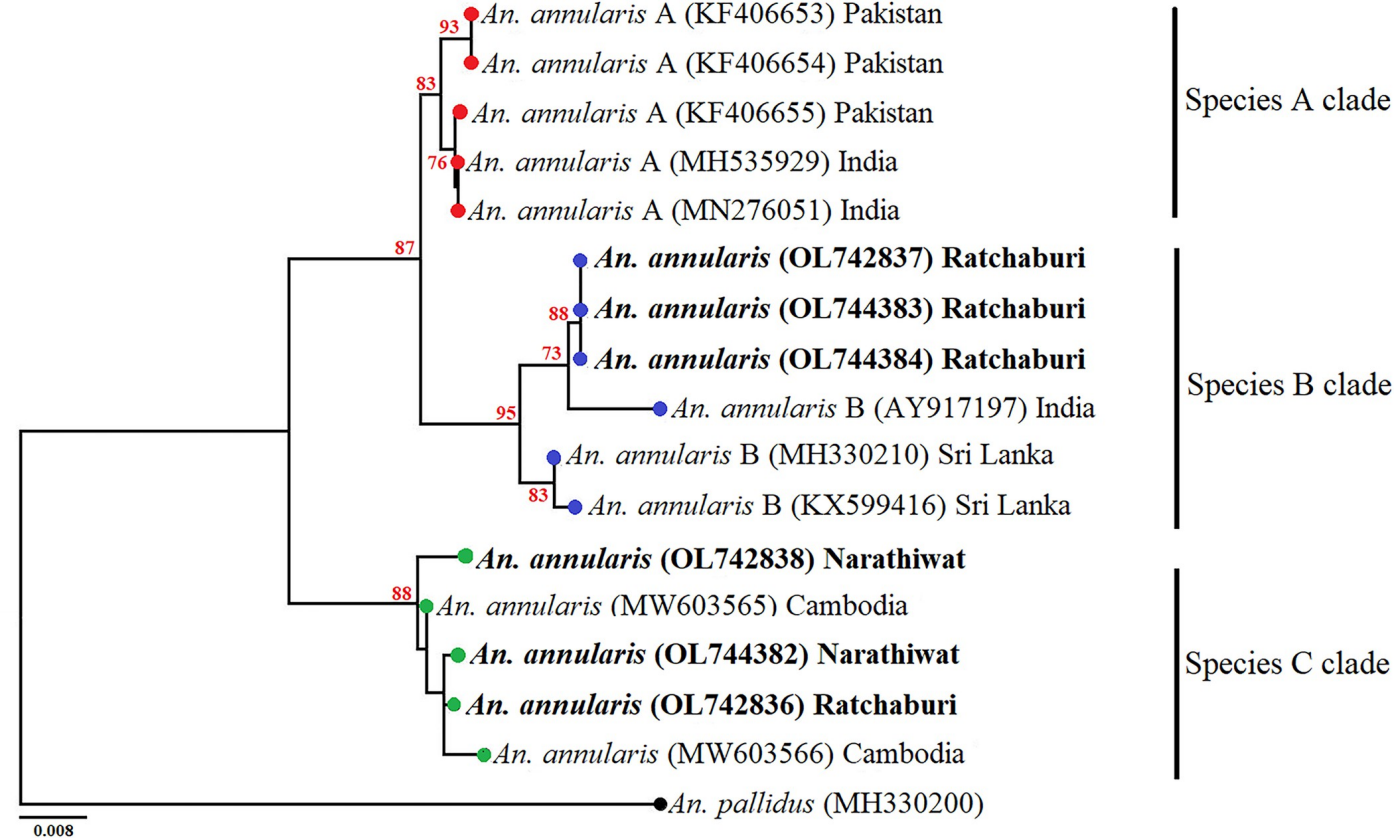
Maximum likelihood (ML) tree based on cytochrome *c* oxidase subunit I (*COI*) sequences of *Anopheles annularis* from this study and their sibling species from GenBank. This tree was constructed using the Tamura 3-parameter substitution model with gamma distribution. Bootstrap values (1000 replicates) are shown near each branch (numbers in red). *Anopheles pallidus* (MH330200) was used as an outgroup to root the tree. The bold font is samples in this study.

**Fig 7:**
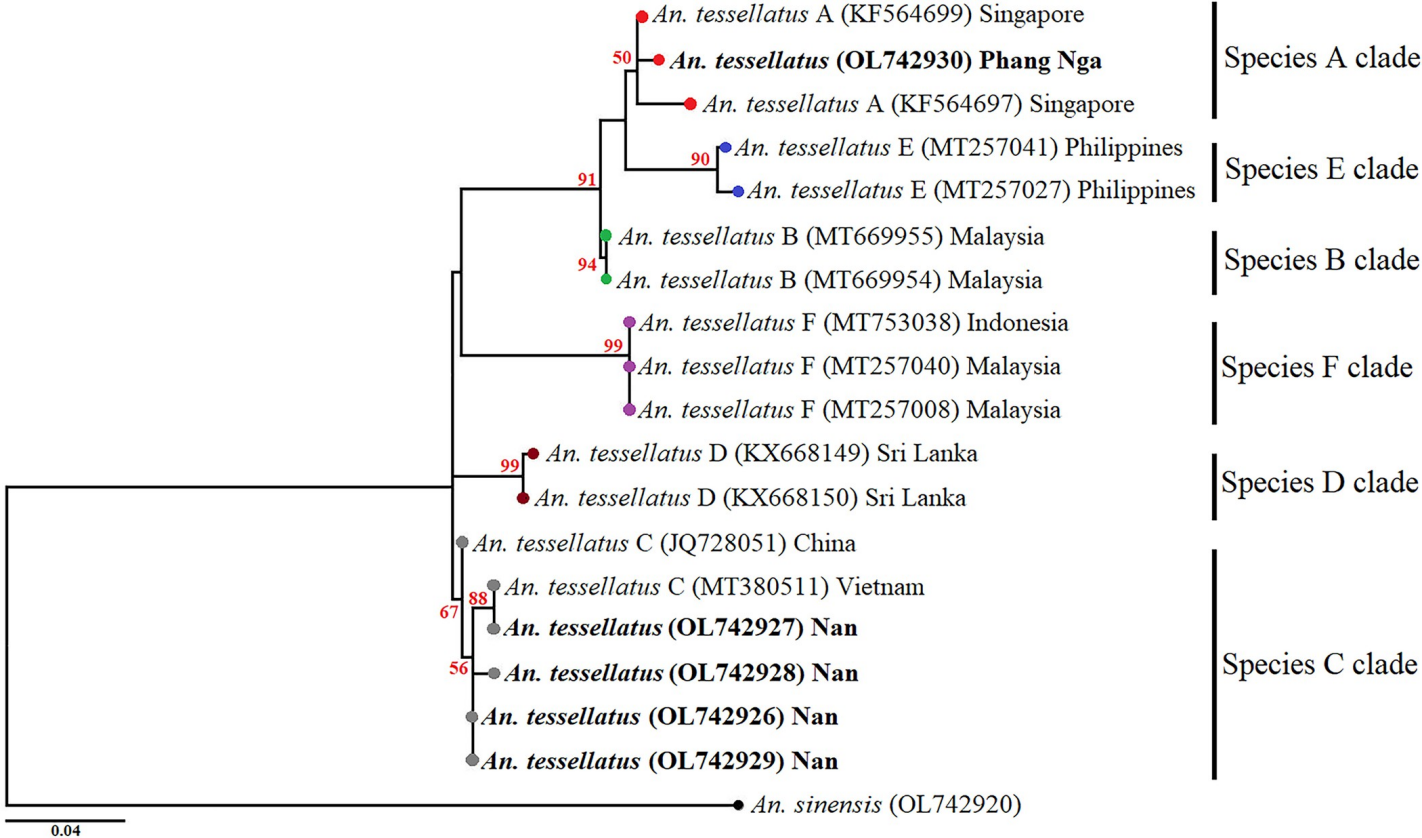
Maximum likelihood (ML) tree based on cytochrome oxidase subunit I (*COI*) sequences of *Anopheles tessellatus* from this study and their sibling species for GenBank. This tree was constructed using the Tamura 3-parameter substitution model with gamma distribution. Bootstrap values (1000 replicates) are shown near each branch (numbers in red). *Anopheles sinensis* (OL742920) was used as an outgroup to root the tree. The bold font is samples in this study.

**Fig 8:**
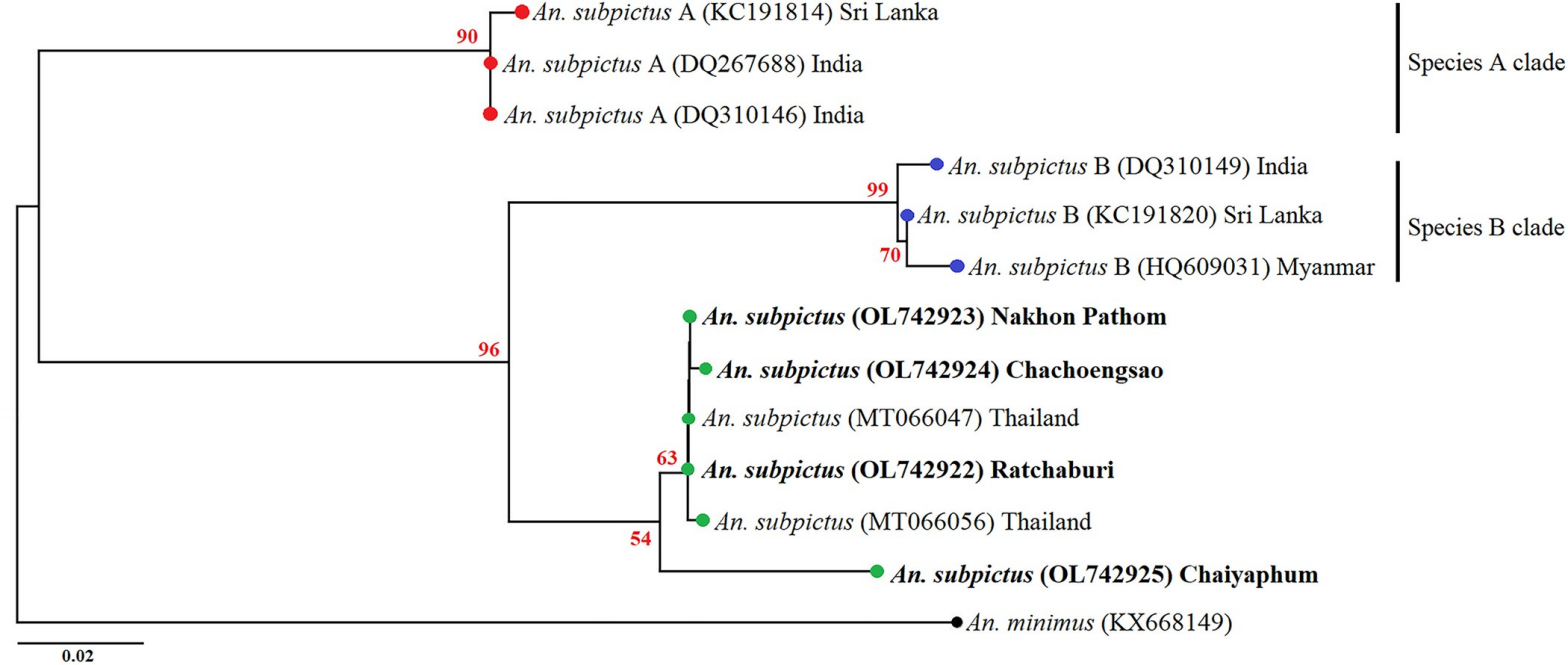
Maximum likelihood (ML) tree based on cytochrome *c* oxidase subunit I (*COI*) sequences of *Anopheles subpictus* from this study and their sibling species for GenBank. This tree was constructed using the Tamura 3-parameter substitution model with gamma distribution. Bootstrap values (1000 replicates) are shown near each branch (numbers in red). *Anopheles minimus* (KX668149) was used as an outgroup to root the tree. The bold font is samples in this study.

BIN analysis indicated that *An*. *annularis*, *An*. *tessellatus*, and *An*. *subpictus* could be different cryptic species in our samples collected because they were clustered into two BINs. Therefore, we compared their sequences with published *COI* sequences from the GenBank database (Figs 6–8). The ML-based phylogenetic analysis of the *An*. *annularis* complex revealed that the three specimens from Ratchaburi province, Western Thailand (OL744383-84 and OL742837), were the closest to *An*. *annularis* species B from Sri Lanka (MH330210 and KX599416) and India (AY917197), whereas one specimen from Ratchaburi province, western Thailand (OL742836), and two specimens from Narathiwat province, Southern Thailand (OL74382 and OL72838), were separated from *An*. *annularis* species A and B (Fig 6). Phylogenetic analyses of the *An*. *tessellatus* complex revealed that four of our specimens from Nan province, Northern Thailand (OL742926-29), were the closest to *An*. *tessellatus* species C from China (JQ728051) and Vietnam (MT380511) (Fig 7). By contrast, one specimen from Phang Nga province, Southern Thailand (OL742930), was the closest to *An*. *tessellatus* species A from Singapore (KF564697 and KF564699). The phylogenetic tree of the *An*. *subpictus* complex revealed that all our specimens were separated from the *An*. *subpictus* species A in India (DQ267688 and DQ310146) and Sri Lanka (KC191814) and species B in India (DQ310149), Sri Lanka (KC191820), and Myanmar (HQ609031) (Fig 8).

## Discussion

The correct identification of mosquito species is important for choosing the optimal vector control approach for the target mosquito species [9]. This study represents a comprehensive mosquito survey in Thailand to compile the *COI* barcode data in an international reference library. We analyzed 310 *COI* nucleotide sequences of mosquitoes, classified into 73 species in 12 genera. Our results provide evidence supporting DNA barcoding as a genetic approach for identifying mosquito species in Thailand.

Our DNA barcode sequences of mosquito specimens had 68.4% AT-richness on average, which is consistent with the results of other studies describing the AT-richness of *COI* sequence in mosquitoes [36,59]. Phylogenetic analyses revealed that almost all mosquito species (71 of 73 species) in the subfamilies Anophelinae and Culicinae were classified according to morphologically identified species (bootstrap support > 97%). *COI* sequence differences at the nucleotide level between groups were used to identify distinct mosquito species in studies from several countries, including Canada [60], China [36], Pakistan [37], Singapore, Sri Lanka [38], the United Kingdom [61], and Mexico [62]. However, in this study, we grouped *An*. *baimaii* and *An*. *dirus* genetically into the same clade. This was consistent with the results from BIN assignment. Similarly, previous studies have reported that nucleotide sequences of *An*. *baimaii* and *An*. *dirus COI* exhibit low interspecific differences [63,64]. Chaiphongpachara et al. [65] recently used DNA barcoding to separate *An*. *dirus* and *An*. *baimaii* in the Thai-Cambodia border, and resulted in the same failure based on low interspecific differences for the mtDNA *COI* gene. They described both *Anopheles* as sibling species which are genetically close species.

In addition, the results of BOLD identification engine based on the comparison with available *COI* sequences in the public database revealed that *Ae*. *vittatus* and *Ae*. *cogilli* were unsuccessfully identified by DNA barcoding because of overlap between genetic distances of the *Ae*. *vittatus* and *Ae*. *cogilli* sequences. Similarly, Díez-Fernández et al. [66] recently reported that both *Aedes* mosquitoes had low interspecific differences in their *COI* nucleotide sequences. However, both species could be distinguished based on their morphology using available taxonomic keys and *Ae*. *cogilli* has not been reported in Thailand.

For *COI* sequence-based species delimitation, the BIN-RESL algorithm method was more accurate than the ASAP method. The results of the BIN-RESL algorithm method was almost all consistent with those of the ML tree, except for those between *Lt*. *fuscana* and *Lt*. *chiangmaiensis*. By contrast, the ASAP method does not efficiently distinguish between species members of groups that are genetically close, including the Minimus complex (*An*. *minimus* and *An*. *harrisoni*), Nigerrimus subgroup (*An*. *nitidus* and *An*. *pursati*), Barbirostris complex (*An*. *dissidens*, *An*. *wejchoochotei*, and *An*. *saeungae*), and *Lutzia* genera (*Lt*. *fuscana* and *Lt*. *chiangmaiensis*). However, more than one method must be used for species delimitation, because each method has its own advantages [56]. The BIN-RESL algorithm method involves clustering based on the assignment of OTUs and putative species from sequence data using RESL [53], whereas ASAP in this study was based on a simple distance (*p*-distances) substitution model that uses threshold values to distinguish between inter- and intraspecific divergence for building species partitions from barcode data sets [56]. However, we checked our *COI* sequences with other substitution models and found that the Jukes-Cantor (JC69) model gave better results (see the S1 Fig). This Jukes-Cantor is a DNA substitution model, in which each base is substituted by any other at an equal rate [67]. Thus, Jukes-Cantor (JC69) model for ASAP can be used to distinguish mosquitoes that are genetically close species such as *Lt*. *chiangmaiensis*, *Lt*. *fuscana*, *An*. *minimus*, *An*.*harrisoni*, *An*.*wejchoochotei*, *An*.*saeungae* and *An*.*dissidens*.

The investigation results of *Lt*. *fuscana* and *Lt*. *chiangmaiensis* by the BIN-RESL algorithm and ASAP methods were not consistent with those of the ML tree. Because their *COI* nucleotide sequences were low interspecific differences (the distance to NN = 1.6%). Similarly, a previous study in Thailand reported that nucleotide sequences of *Lt*. *fuscana* and *Lt*. *chiangmaiensis COI* exhibited low interspecific variations (genetic distances = 0.2–2.4%) [68]. However, ML phylogenetic analysis can assist in the assessment of both species based on this study. Thus, phylogenetic analysis remains critical in species assessment and should be applied to DNA barcoding.

Although the results of the identification success rates based on “Best Match,” “Best Close Match,” and “All Species Barcodes” of our *COI* barcode dataset revealed quite high values, intra- and interspecific genetic divergence showed the absence of the “barcoding gap” between mosquito species because of the slight overlap between *An*. *baimaii* and *An*. *dirus* (the distance to NN is less than the maximum intraspecific distance [55]). The barcode gap, a hiatus between *COI* intraspecific and interspecific genetic distances, is important for DNA barcoding and is effective in species identification [69,70]. Several studies have shown the absence of the barcode gap in mosquitoes found in very close species or species complexes, containing closely related taxa [35,70,71]. However, finding two mosquito species with an overlap between intra- and interspecific divergence values does not mean that DNA barcoding failed to identify mosquito species, with error accounting for only 2.7% (2 out of 73 species).

Furthermore, *An*. *paraliae* samples were classified as *An*. *lesteri* based on BIN assignment. This contradiction can be explained on the basis of a study by Taai et al. [72], who reported that both *Anopheles* mosquito species could represent the same species based on crossing experiments, morphological variations, and genetic relationships, which are based on second internal transcribed spacer (ITS2) of ribosomal DNA and *COI* and *COII* of mitochondrial DNA [72]. The taxonomic changes in mosquitoes are frequently due to the discovery of new biological evidence, such as morphological and genetic evidence [1]. In this study, new scientific names of three mosquito species, namely, *Ochlerotatus vigilax*, *Rh*. *longirostris*, and *Co*. *macfarlanei* were used [1]. However, the BOLD database remains unmodified, with all three mosquito species still *Aedes vigilax*, *Aedes longirostris*, and *Aedes macfarlanei*. Therefore, BOLD users should have basic taxonomic knowledge to avoid confusion.

For species investigation, DNA barcoding cannot separate mosquito species with low/no interspecific differences in their *COI* nucleotide sequences. However, several studies have reported that DNA barcoding can reveal the cryptic species of some mosquito complexes [62,71]. Usually, insects display approximately ≤2% intraspecific genetic divergence [71,73,74]. The assessment of *COI* nucleotide sequence difference within species showed that mosquito species with the high intraspecific divergence (>2%) included *An*. *annularis* (2.6%), with two BINs (BOLD: AAR3272 and AEG7105) and *An*. *tessellatus* (2.5%) with two BINs (BOLD: AAT9116 and ACW0305). Additionally, ML tree analysis of mosquito species in the subfamily Anophelinae showed subgroup separations in *An*. *annularis*, *An*. *tessellatus*, and *An*. *subpictus* groups. Subsequently, phylogenetic analyses of our *An*. *annularis* and *An*. *tessellatus* specimens against previous *COI* sequences of sibling species within their species complexes revealed that they were potentially composed of two sibling species within both species groups.

Currently, the *An*. *annularis* complex contains two sibling species A and B [75]. In this study, phylogenetic analysis revealed that the three *An*. *annularis* specimens correspond to *An*. *annularis* species B, whereas the other three specimens were distinct from species A and B. It is possible that it may be a new *An*. *annularis* species “C”. However, sibling species assessment at this time is preliminary. Further investigations must be performed in detail using several DNA markers, such as ITS2 16S rDNA and 18S rDNA [76]. *Anopheles annularis* is an important malaria vector in Nepal and Bangladesh and a secondary malaria vector in India and Sri Lanka [77]. In Thailand, *An*. *annularis* is an important malaria vector in the Tak province, based on the detection of both *Plasmodium falciparum* and *P*. *vivax* in this mosquito species [7]. However, the sibling species of this complex investigation in several countries remains unclear, except for those in India, Sri Lanka, and Pakistan [77].

A recent study reported that the *An*. *tessellatus* complex includes at least six sibling species: A, B, C, D, E, and F [78]. In addition, these species can transmit *Plasmodium* parasites in Sumatra, Indonesia [79], and Sri Lanka [80]. Our analysis of phylogenetic relationships showed that the specimens correspond to *An*. *tessellatus* species A and C. Specimens from Nan province, Northern Thailand, were grouped into *An*. *tessellatus* species C, which is consistent with previous studies that described that they are widely spread in mainland Southeast Asia, such as China and Laos, a territory near Northern Thailand [78]. One specimen from the Phang Nga province, Southern Thailand, was grouped into *An*. *tessellatus* species A, which is consistent with previous studies that found that this species was distributed in Singapore, where it was nearly located in the Southern region of Thailand [78].

Likewise, *An*. *subpictus* was investigated because this mosquito is reported to be species complex. The *An*. *subpictus* complex contain four sibling species: A, B, C, and D [75]. In this study, phylogenetic analyses revealed that no *An*. *subpictus* specimen belonged to species A or B. Consistently, a study reported that *An*. *subpictus* is distinct from species A and B in Thailand [81]. We could not assess the sibling species of the *An*. *Subpictus* complex, because *COI* or ITS2 can be used to identify only species A and B [81]. Therefore, we recommend performing crossing experiments and cytogenetic analysis, which were used to identify mosquito species in this complex [77].

## Conclusions

Our results confirm that DNA barcoding is an effective molecular approach for the accurate identification of mosquitoes in Thailand. However, some mosquitoes that are genetically closely related cannot be identified using this technique based on our findings on *An*. *baimaii* and *An*. *dirus*. Other modern techniques are needed to support true species identification of two *Anopheles* mosquitoes. We submitted all *COI* nucleotide sequences of the mosquitoes obtained in this study to the GenBank and BOLD databases. Our results can be used for species identification and investigating genetic variations related to the geographical distribution of mosquito vectors. Furthermore, this study is the first to report the *COI* sequences of four mosquito species: *An*. *pseudojamesi*, *Co*. *macfarlanei*, *Mi*. *aurea*, and *Rh*. *longirostris*, which were added to the GenBank and BOLD databases.

## Supporting information

S1 Fig Result of ASAP based on Jukes-Cantor (JC69) model. (PDF)

S1 Table Detailed data of mosquito specimens. (PDF)
