# Correction: Mitochondrial DNA barcoding of mosquito species (Diptera: Culicidae) in Thailand

**DOI:** 10.1371/journal.pone.0317172

**Published:** 2025-01-03

**Authors:** The PLOS ONE Staff

There are errors in Table 2 and Table 3. Please see the complete, correct Table 2 and Table 3 here.

**Table 2:** Barcode Index Number details and genetic distance to the nearest species (minimum interspecific distance) based on barcode gap analysis in the Barcode of Life Data Systems.

Mosquito species	Barcode Index Number (BIN) details			The nearest species	Distance to the nearest neighbour
	Barcode index numbers ID	Total members	Count in project		
*Ad*. *catasticta*	ACV6316	5	4	*Lt*. *chiangmaiensis*	10.6
*Ae*. *aegypti*	AAA4210	2193	4	*Ae*. *vexans*	9.2
*Ae*. *albopictus*	AAA5870	3776	5	*Ae*. *lineatopennis*	10.2
*Ae*. *desmotes*	ADK1864	32	2	*Ae*. *vittatus*	9.5
*Ae*. *lineatopennis*	ACB9609	12	4	*Ae*. *vittatus*	7.6
*Ae*. *poicilius*	AEK4068	3	2	*Cx*. *sitiens*	10.4
*Ae*. *vexans*	AAA7067	4873	7	*Ae*. *vittatus*	8.1
*Ae*. *vittatus*	AAV4160	120	5	*Ae*. *lineatopennis*	7.6
*An*. *aconitus*	AAB2519	42	5	*An*. *minimus*	8.1
*An*. *annularis*	AAR3272	91	3	*An*. *culicifacies*	7.6
	AEG7105	8	3		
*An*. *baimaii*	ABZ2357	24	2	*An*. *dirus*	0.3
*An*. *culicifacies*	AAF0681	40	2	*An*. *minimus*	7.1
*An*. *dirus*	AAC7100	11	3	*An*. *baimaii*	0.3
	ABZ2357	24	3		
*An*. *dissidens*	AAA5684	142	2	*An*. *saeungae*	3.3
*An*. *dravidicus*	ABY9593	14	6	*An*. *maculatus*	6.3
*An*. *epiroticus*	AAA4214	169	5	*An*. *culicifacies*	9.5
*An*. *harrisoni*	ACF2761	49	3	*An*. *minimus*	3.0
*An*. *jamesii*	AAD2562	43	4	*An*. *maculatus*	8.8
*An*. *maculatus*	AAC3408	39	5	*An*. *dravidicus*	6.3
*An*. *minimus*	AAB5964	39	5	*An*. *harrisoni*	3.0
*An*. *nemophilous*	AEA5944	23	1	*An*. *dirus*	5.6
*An*. *nigerrimus*	AAI4557	22	1	*An*. *pursati*	5.8
*An*. *nitidus*	AAA5749	53	3	*An*. *pursati*	4.0
*An*. *nivipes*	AAD9018	37	2	*An*. *philippinensis*	7.3
*An*. *paraliae*	AAA5748	113	6	*An*. *peditaeniatus*	4.1
*An*. *peditaeniatus*	AAD3070	163	1	*An*. *paraliae*	4.1
*An*. *philippinensis*	ADS6721	5	4	*An*.*nivipes*	7.3
*An*. *pseudojamesi*	AEQ7311	6	6	*An*. *philippinensis*	8.5
*An*. *pseudowillmori*	AAJ2796	18	2	*An*. *dirus*	9.3
*An*. *pursati*	ACQ5333	19	3	*An*. *nitidus*	4.0
*An*. *saeungae*	ACF3961	11	2	*An*. *wejchoochotei*	1.9
*An*. *sawadwongporni*	AAC3407	44	6	*An*. *maculatus*	6.8
*An*. *sinensis*	AAA5339	726	2	*An*. *paraliae*	4.3
*An*. *subpictus*	AAA4215	20	3	*An*. *paraliae*	9.5
	ABY5601	10	1		
*An*. *tessellatus*	AAT9116	46	4	*An*. *saeungae*	7.8
	ACW0305	5	1		
*An*. *vagus*	AAE4158	63	3	*An*. *paraliae*	10.1
*An*. *varuna*	AAD5962	19	2	*An*. *culicifacies*	8.2
*An*. *wejchoochotei*	AAA5682	84	8	*An*. *saeungae*	1.9
*Ar*. *durhami*	ADU2633	13	6	*Ar*. *subalbatus*	9.1
*Ar*. *flavus*	ACP1995	24	2	*Ar*. *malayi*	9.9
*Ar*. *malayi*	ADT8626	2	1	*Ar*. *subalbatus*	9.4
*Ar*. *subalbatus*	AAC8113	428	5	*Ae*. *lineatopennis*	8.2
*Co*. *macfarlanei*	AEP6081	4	4	*Ae*. *vigilax*	9.5
*Cq*. *crassipes*	AAU6779	15	4	*Cq*. *ochracea*	11.7
*Cq*. *ochracea*	ADJ5998	9	6	*Co*. *macfarlanei*	10.3
*Cx*. *bicornutus*	ACC8728	25	8	*Cx*. *epidesmus*	8.3
*Cx*. *bitaeniorhynchus*	AAJ7281	100	4	*Cx*. *epidesmus*	4.6
*Cx*. *brevipalpis*	ACC8753	8	4	*Cq*. *ochracea*	10.6
*Cx*. *epidesmus*	AAZ7107	10	3	*Cx*. *bitaeniorhynchus*	4.6
*Cx*. *fuscocephala*	AAJ7295	51	5	*Cx*. *epidesmus*	6.6
*Cx*. *gelidus*	AAC6669	157	6	*Cx*. *sitiens*	5.3
*Cx*. *infantulus*	AAZ3192	14	8	*Cx*. *gelidus*	6.6
*Cx*. *murrelli*	ACC8692	11	6	*Cx*. *bitaeniorhynchus*	6.3
*Cx*. *nigropunctatus*	AAR4910	41	7	*Cx*. *pallidothorax*	4.1
*Cx*. *pallidothorax*	AAZ3501	143	7	*Cx*. *nigropunctatus*	4.1
*Cx*. *pseudovishnui*	ACM5555	14	2	*Cx*. *vishnui*	4.5
*Cx*. *quinquefasciatus*	AAA4751	6459	8	*Cx*. *pseudovishnui*	7.3
*Cx*. *sitiens*	AAB7378	189	7	*Cx*. *gelidus*	5.3
*Cx*. *tritaeniorhynchus*	AAE3201	2716	6	*Cx*. *vishnui*	4.8
*Cx*. *vishnui*	AAI9879	41	7	*Cx*. *pseudovishnui*	4.5
*Lt*. *vorax*	ACC8609	43	2	*Lt*. *fuscana*	4.5
*Lt*. *fuscana*	AAG3834	69	6	*Lt*. *chiangmaiensis*	1.6
*Lt*. *chiangmaiensis*	AAG3834	69	6	*Lt*. *fuscana*	1.6
*Ma*. *annulifera*	AAF2325	27	6	*Ma*. *bonneae*	12.3
*Ma*. *bonneae*	AAO3240	48	5	*Ma*. *dives*	4.8
*Ma*. *dives*	ADS1936	43	2	*Ma*. *bonneae*	4.8
*Ma*. *indiana*	AAC8223	24	3	*Ma*. *bonneae*	11.1
*Ma*. *uniformis*	AAB7890	93	6	*Ma*. *bonneae*	9.7
*Mi*. *aurea*	AEP3699	3	3	*Lt*. *chiangmaiensis*	12.4
*Oc*. *vigilax*	AAC1707	250	4	*Ae*. *lineatopennis*	9.5
*Rh*. *longirostris*	AEP6082	2	2	*Ae*. *lineatopennis*	9.0
*Tx*. *splendens*	AAC7483	13	2	*Ae*. *poicilius*	12.9
*Ur*. *obscura*	AEI3994	4	2	*Ae*. *poicilius*	12.0

**Table 3:** Results of BOLD identification engine.

Mosquito species in present study	*n*	Average match (%)	Min–Max (%)	Match species in Bold database	Match sequence number from queries to the BOLD ID engine
*Ad*. *catasticta*	4	98.3	97.0–99.5	*Ad*. *catasticta*	6
*Ae*. *aegypti*	4	99.6	98.5–100	*Ae*. *aegypti*	100
*Ae*. *albopictus*	5	99.9	99.8–100	*Ae*. *albopictus*	101
*Ae*. *desmotes*	2	99.0	97.0–99.7	*Ae*. *desmotes*	13
*Ae*. *lineatopennis*	4	98.6	97.6–99.8	*Ae*. *lineatopennis*	10
*Ae*. *poicilius*	2	99.6	99.1–100	*Ae*. *poicilius*	2
*Ae*. *vexans*	7	97.8	96.7–100	*Ae*. *vexans*	100
*Ae*. *vittatus*	5	98.4	97.8–99.8	*Ae*. *vittatus*	53
		98.4	97.6–99.1	*Ae*. *cogilli*	47
*An*. *aconitus*	5	97.9	95.21–99.8	*An*. *aconitus*	73
*An*. *annularis*	6	97.0	93.3–100	*An*. *annularis*	99
*An*. *baimaii*	2	99.2	99.2–99.2	*An*. *baimaii*	2
		98.8	97.4–100	*An*. *dirus*	35
*An*. *culicifacies*	2	97.8	94.8–99.8	*An*. *culicifacies*	48
*An*. *dirus*	6	98.7	97.3–100	*An*. *dirus*	34
		99.3	98.9–99.7	*An*. *baimaii*	2
*An*. *dissidens*	2	99.6	99.6–99.8	*An*. *dissidens*	58
*An*. *dravidicus*	6	98.8	96.5–99.8	*An*. *dravidicus*	14
*An*. *epiroticus*	5	97.9	96.8–100	*An*. *epiroticus*	34
*An*. *harrisoni*	3	99.1	98.5–99.6	*An*. *harrisoni*	13
*An*. *jamesii*	4	99.0	96.3–99.8	*An*. *jamesii*	22
*An*. *maculatus*	5	97.6	93.0–100	*An*. *maculatus*	70
*An*. *minimus*	5	98.2	96.5–100	*An*. *minimus*	81
*An*. *nemophilous*	1	97.7	97.4–98.1	*An*. *introlatus*	21
*An*. *nigerrimus*	1	98.6	95.3–99.6	*An*. *nigerrimus*	23
*An*. *nitidus*	3	99.0	97.6–100	*An*. *nitidus*	46
*An*. *nivipes*	2	98.9	98.5–99.6	*An*. *nivipes*	36
*An*. *paraliae*	6	98.4	97.4–99.8	*An*. *lesteri*	99
*An*. *peditaeniatus*	1	99.6	99.3–100	*An*. *peditaeniatus*	100
*An*. *philippinensis*	4	98.0	95.2–99.9	*An*. *philippinensis*	5
*An*. *pseudowillmori*	2	99.3	98.5–100	*An*. *pseudowillmori*	16
*An*. *pursati*	3	98.6	98.0–99.4	*An*. *pursati*	18
*An*. *saeungae*	2	98.8	97.6–99.7	*An*. *saeungae*	24
*An*. *sawadwongporni*	6	99.4	98.7–99.8	*An*. *sawadwongporni*	22
*An*. *sinensis*	2	98.8	98.7–99.5	*An*. *sinensis*	86
*An*. *subpictus*	4	96.1	91.1–100	*An*. *subpictus*	19
*An*. *tessellatus*	5	96.4	92.1–99.8	*An*. *tessellatus*	96
*An*. *vagus*	3	98.3	95.6–100	*An*. *vagus*	48
*An*. *varuna*	2	99.2	98.1–99.8	*An*. *varuna*	17
*An*. *wejchoochotei*	8	99.5	98.5–100	*An*. *wejchoochotei*	46
*Ar*. *durhami*	6	99.7	99.2–100	*Ar*. *durhami*	12
*Ar*. *flavus*	2	98.8	98.1–99.8	*Ar*. *flavus*	5
*Ar*. *malayi*	1	99.6	99.6–99.6	*Ar*. *malayi*	1
*Ar*. *subalbatus*	5	99.9	99.8–100	*Ar*. *subalbatus*	100
*Cq*. *crassipes*	4	98.4	96.4–99.6	*Cq*. *crassipes*	16
*Cq*. *ochracea*	6	99.4	98.5–99.4	*Cq*. *ochracea*	8
*Cx*. *bicornutus*	8	99.3	98.25–100	*Cx*. *bicornutus*	13
*Cx*. *bitaeniorhynchus*	4	98.9	97.4–100	*Cx*. *bitaeniorhynchus*	73
*Cx*. *brevipalpis*	4	97.0	94.4–100	*Cx*. *brevipalpis*	23
*Cx*. *epidesmus*	3	99.2	98.5–99.8	*Cx*. *epidesmus*	8
*Cx*. *fuscocephala*	5	99.5	98.2–100	*Cx*. *fuscocephala*	48
*Cx*. *gelidus*	6	99.1	98.1–100	*Cx*. *gelidus*	100
*Cx*. *infantulus*	8	98.0	96.1–99.8	*Cx*. *infantulus*	28
*Cx*. *murrelli*	6	98.9	96.1–99.8	*Cx*. *murrelli*	9
*Cx*. *nigropunctatus*	7	98.4	96.5–99.7	*Cx*. *nigropunctatus*	32
*Cx*. *pallidothorax*	7	98.9	98.3–99.5	*Cx*. *pallidothorax*	99
*Cx*. *pseudovishnui*	2	96.9	95.2–99.8	*Cx*. *pseudovishnui*	50
*Cx*. *quinquefasciatus*	8	99.9	99.8–100	*Cx*. *quinquefasciatus*	35
*Cx*. *sitiens*	7	99.5	98.7–100	*Cx*. *sitiens*	98
*Cx*. *tritaeniorhynchus*	6	99.6	99.2–100	*Cx*. *tritaeniorhynchus*	100
*Cx*. *vishnui*	7	99.2	95.2–100	*Cx*. *vishnui*	38
*Lt*. *vorax*	2	98.8	97.8–99.8	*Lt*. *vorax*	36
*Lt*. *fuscana*	6	99.0	96.9–100	*Lt*. *fuscana*	34
*Lt*. *chiangmaiensis*	6	99.4	98.3–100	*Lt*. *chiangmaiensis*	15
*Ma*. *annulifera*	6	98.1	96.7–99.2	*Ma*. *annulifera*	26
*Ma*. *bonneae*	5	98.9	98.3–99.8	*Ma*. *bonneae*	21
*Ma*. *dives*	2	97.6	96.6–99.5	*Ma*. *dives*	16
*Ma*. *indiana*	3	99.2	98.7–100	*Ma*. *indiana*	23
*Ma*. *uniformis*	6	98.6	95.7–99.8	*Ma*. *uniformis*	80
*Oc*. *vigilax*	4	99.1	98.7–99.8	*Oc*. *vigilax*	100
*Tx*. *splendens*	2	98.2	97.1–100	*Tx*. *splendens*	32
*Ur*. *obscura*	2	99.4	98.9–100	*Ur*. *obscura*	3

Four species including *An*. *pseudojamesi*, *Co*. *macfarlanei*, *Mi*. *aurea*, and *Rh*. *longirostris* as new records are not included in the table.

The publisher apologizes for the errors.
