# Supplementary figures and images for: Mitochondrial DNA barcoding of mosquito species (Diptera: Culicidae) in Thailand
Source: PLoS One. 2022 Sep 22;17(9):e0275090. doi: 10.1371/journal.pone.0275090 (PMC9642330; doi:10.1371/journal.pone.0275090)

# Supplementary Fig. S1

## Result of ASAP based on Jukes-Cantor (JC69) model.

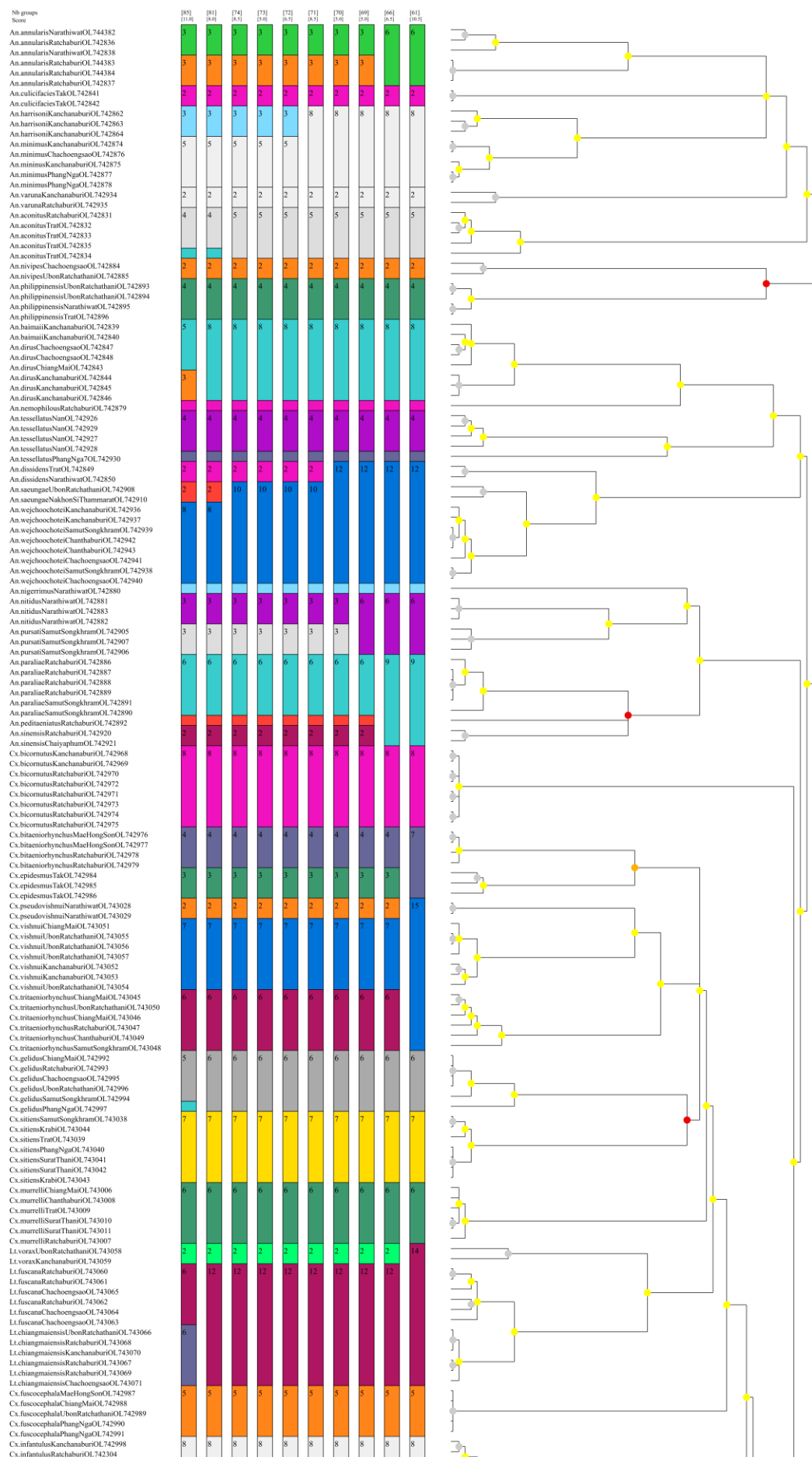

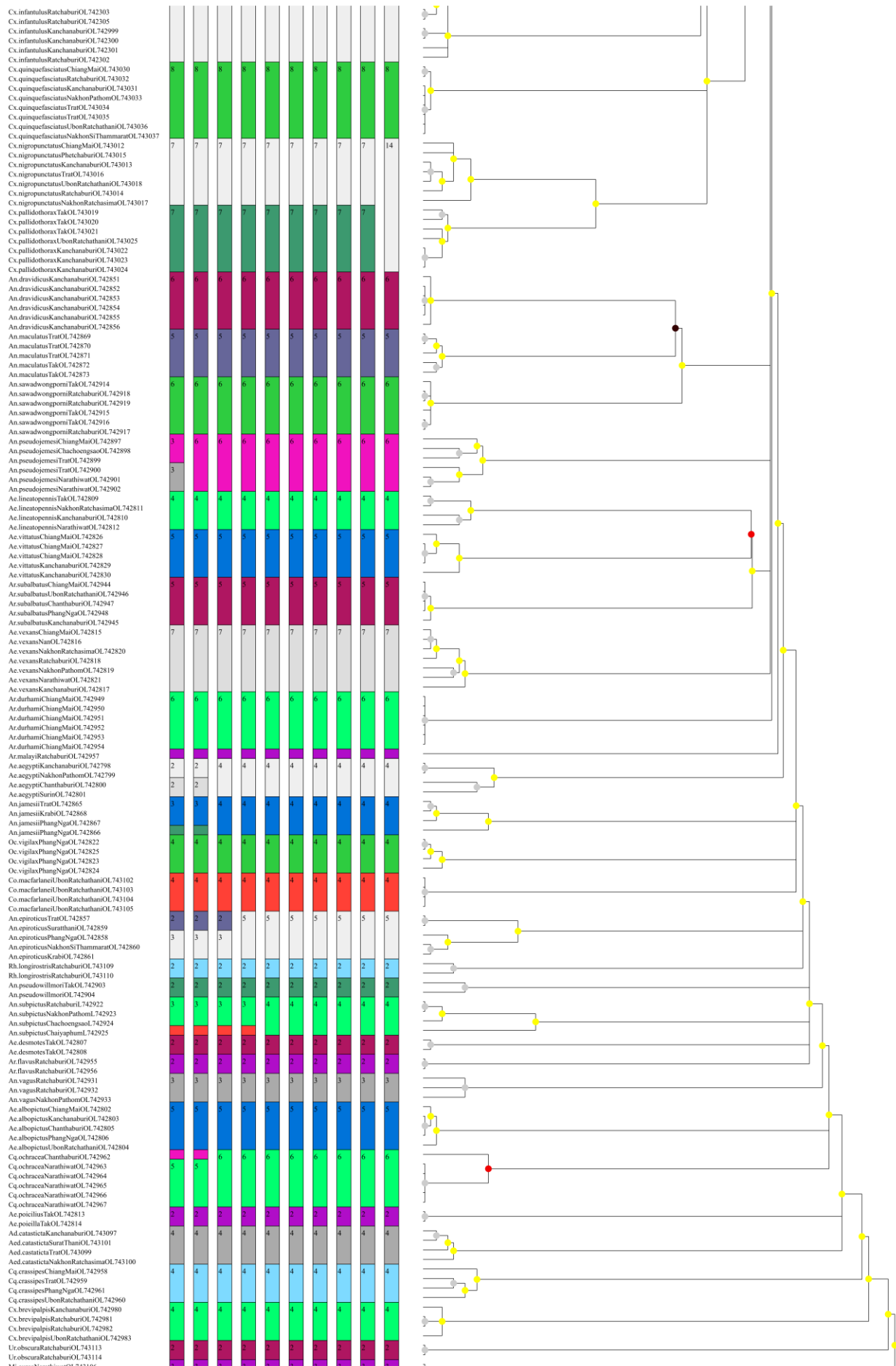

Supplement: S1 Fig — (PDF) [file pone.0275090.s001.pdf]
