# Supplementary material for: Mitochondrial DNA barcoding of mosquito species (Diptera: Culicidae) in Thailand
Source: PLoS One. 2022 Sep 22;17(9):e0275090. doi: 10.1371/journal.pone.0275090 (PMC9642330; doi:10.1371/journal.pone.0275090)
Supplement: S1 Table — (PDF) [file pone.0275090.s002.pdf]

Table S1. Detailed data of mosquito specimens.

	Species	Specimen voucher	GenBank accession numbers	Collection	Province	Geographical region	Latitude/ longitude
1	*Aedes aegypti*	4814	OL742798	Adult mosquito trapping	Kanchanaburi	Western	14°06'37.5"N 98°59'52.9"E
2	*Aedes aegypti*	2955.1	OL742799	Adult mosquito trapping	Nakhon Pathom	Central	13°53'29.6"N 100°00'08.8"E
3	*Aedes aegypti*	6365.1	OL742800	Adult mosquito trapping	Chanthaburi	Eastern	13°22'43.2"N 99°16'23.9"E
4	*Aedes aegypti*	6327.1	OL742801	Larvae dipper	Surin	Northeastern	14°37'23.7"N 103°53'55.6"E
5	*Aedes albopictus*	6609.1	OL742801	Larvae dipper	Chiang Mai	Northern	19°10'26.4"N 99°01'49.2"E
6	*Aedes albopictus*	5157.1	OL742802	Larvae dipper	Kanchanaburi	Western	14°14'30.3"N 99°20'21.5"E
7	*Aedes albopictus*	6315.1	OL742803	Adult mosquito trapping	Ubon Ratchathani	Northeastern	14°26'54.1"N 105°12'33.0"E
8	*Aedes albopictus*	5323.1	OL742804	Larvae dipper	Chanthaburi	Eastern	12°51'29.8"N 102°16'32.0"E
9	*Aedes albopictus*	7250.1	OL742805	Adult mosquito trapping	Phang Nga	Southern	8°28'06.5"N 98°35'19.6"E
10	*Aedes desmotes*	1209	OL742807	Adult mosquito trapping	Tak	Western	17°33'26.2"N 97°55'15.8"E
11	*Aedes desmotes*	1214	OL742808	Adult mosquito trapping	Tak	Western	17°33'26.2"N 97°55'15.8"E
12	*Aedes lineatopennis*	1243	OL742809	Adult mosquito trapping	Tak	Western	17°33'26.2"N 97°55'15.8"E
13	*Aedes lineatopennis*	6816	OL742810	Adult mosquito trapping	Kanchanaburi	Western	14°07'06.2"N 99°01'16.2"E
14	*Aedes lineatopennis*	6043.1	OL742811	Adult mosquito trapping	Nakhon Ratchasima	Northeastern	14°30'56.4"N 101°22'31.7"E
15	*Aedes lineatopennis*	4210.1	OL742812	Adult mosquito trapping	Narathiwat	Southern	6°21'19.3"N 101°53'41.6"E
16	*Aedes poicilius*	1207	OL742813	Adult mosquito trapping	Tak	Western	17°33'26.2"N 97°55'15.8"E
17	*Aedes poicilius*	1208	OL742814	Adult mosquito trapping	Tak	Western	17°33'26.2"N 97°55'15.8"E
18	*Aedes vexans*	6433.18	OL742815	Adult mosquito trapping	Chiang Mai	Northern	18°41'33.9"N 98°55'48.9"E
19	*Aedes vexans*	4446.1	OL742816	Adult mosquito trapping	Nan	Northern	18°43'11.0"N 100°46'04.0"E
20	*Aedes vexans*	7653	OL742817	Adult mosquito trapping	Kanchanaburi	Western	14°07'06.2"N 99°01'16.2"E
21	*Aedes vexans*	6708	OL742818	Adult mosquito trapping	Ratchaburi	Western	13°21'35.3"N 99°15'04.9"E
22	*Aedes vexans*	2975.1	OL742819	Adult mosquito trapping	Nakhon Pathom	Central	13°53'29.6"N 100°00'08.8"E
23	*Aedes vexans*	6071.1	OL742820	Adult mosquito trapping	Nakhon Ratchasima	Northeastern	14°30'56.4"N 101°22'31.7"E
24	*Aedes vexans*	4192.6	OL742821	Adult mosquito trapping	Narathiwat	Southern	6°21'19.3"N 101°53'41.6"E
25	*Aedes vittatus*	6391.1	OL742826	Adult mosquito trapping	Chiang Mai	Northern	18°41'33.9"N 98°55'48.9"E
26	*Aedes vittatus*	6600	OL742827	Larvae dipper	Chiang Mai	Northern	18°51'18.5"N 98°42'07.3"E
27	*Aedes vittatus*	6600.1	OL742828	Larvae dipper	Chiang Mai	Northern	18°51'18.5"N 98°42'07.3"E
28	*Aedes vittatus*	4904.1	OL742829	Adult mosquito trapping	Kanchanaburi	Western	14°07'06.2"N 99°01'16.2"E
29	*Aedes vittatus*	4904.2	OL742830	Adult mosquito trapping	Kanchanaburi	Western	14°07'06.2"N 99°01'16.2"E
30	*Aedeomyia catasticta*	5559.1	OL743097	Adult mosquito trapping	Kanchanaburi	Western	14°07'06.2"N 99°01'16.2"E
31	*Aedeomyia catasticta*	5840.1	OL743099	Adult mosquito trapping	Trat	Eastern	12°20'40.5"N 102°26'58.8"E
32	*Aedeomyia catasticta*	6040.1	OL743100	Adult mosquito trapping	Nakhon Ratchasima	Northeastern	14°30'56.4"N 101°22'31.7"E
33	*Aedeomyia catasticta*	7023.1	OL743101	Adult mosquito trapping	Surat Thani	Southern	9°13'56.9"N 99°14'15.3"E
34	*Anopheles aconitus*	7522	OL742831	Adult mosquito trapping	Ratchaburi	Western	13°22'35.7"N 99°16'37.2"E
35	*Anopheles aconitus*	5729	OL742832	Adult mosquito trapping	Trat	Eastern	12°28'13.6"N 102°41'05.5"E
36	*Anopheles aconitus*	5787.1	OL742833	Adult mosquito trapping	Trat	Eastern	12°28'13.6"N 102°41'05.5"E

	Species	Specimen voucher	GenBank accession numbers	Collection	Province	Geographical region	Latitude/ longitude
37	*Anopheles aconitus*	5814	OL742834	Adult mosquito trapping	Trat	Eastern	12°28'13.6"N 102°41'05.5"E
38	*Anopheles aconitus*	5814.1	OL742835	Adult mosquito trapping	Trat	Eastern	12°28'13.6"N 102°41'05.5"E
39	*Anopheles annularis*	6727.2	OL742836	Larvae dipper	Ratchaburi	Western	13°22'33.7"N 99°16'25.9"E
40	*Anopheles annularis*	6727.3	OL742837	Larvae dipper	Ratchaburi	Western	13°22'33.7"N 99°16'25.9"E
41	*Anopheles annularis*	4461	OL744383	Adult mosquito trapping	Ratchaburi	Western	13°21'35.1"N 99°15'04.5"E
42	*Anopheles annularis*	6727.1	OL744384	Larvae dipper	Ratchaburi	Western	13°22'33.7"N 99°16'25.9"E
43	*Anopheles annularis*	4134.4	OL742838	Adult mosquito trapping	Narathiwat	Southern	6°21'19.3"N 101°53'41.6"E
44	*Anopheles annularis*	5205	OL744382	Adult mosquito trapping	Narathiwat	Southern	6°21'19.3"N 101°53'41.6"E
45	*Anopheles baimaii*	3481	OL742839	Adult mosquito trapping	Kanchanaburi	Western	14°07'06.2"N 99°01'16.2"E
46	*Anopheles baimaii*	5589.3	OL742840	Adult mosquito trapping	Kanchanaburi	Western	14°07'06.2"N 99°01'16.2"E
47	*Anopheles culicifacies*	2619TM	OL742841	Adult mosquito trapping	Tak	Western	17°33'26.2"N 97°55'15.8"E
48	*Anopheles culicifacies*	2619.1TM	OL742842	Adult mosquito trapping	Tak	Western	17°33'26.2"N 97°55'15.8"E
49	*Anopheles dirus*	6468	OL742843	Adult mosquito trapping	Chiang Mai	Northern	18°51'18.5"N 98°42'07.3"E
50	*Anopheles dirus*	5589.1	OL742844	Adult mosquito trapping	Kanchanaburi	Western	14°07'06.2"N 99°01'16.2"E
51	*Anopheles dirus*	5589.2	OL742845	Adult mosquito trapping	Kanchanaburi	Western	14°07'06.2"N 99°01'16.2"E
52	*Anopheles dirus*	6876	OL742846	Adult mosquito trapping	Kanchanaburi	Western	14°07'06.2"N 99°01'16.2"E
53	*Anopheles dirus*	4048.9	OL742847	Adult mosquito trapping	Chachoengsao	Eastern	13°30'16.7"N 101°47'09.2"E
54	*Anopheles dirus*	4048.10	OL742848	Adult mosquito trapping	Chachoengsao	Eastern	13°30'16.7"N 101°47'09.2"E
55	*Anopheles dissidens*	5914.1	OL742849	Adult mosquito trapping	Trat	Eastern	12°22'13.3"N 102°41'00.9"E
56	*Anopheles dissidens*	5209	OL742850	Adult mosquito trapping	Narathiwat	Southern	6°21'19.3"N 101°53'41.6"E
57	*Anopheles dravidicus*	3709.1	OL742851	Adult mosquito trapping	Kanchanaburi	Western	14°07'06.2"N 99°01'16.2"E
58	*Anopheles dravidicus*	4913	OL742852	Larvae dipper	Kanchanaburi	Western	14°14'30.4"N 99°20'21.6"E
59	*Anopheles dravidicus*	4914	OL742853	Larvae dipper	Kanchanaburi	Western	14°14'30.4"N 99°20'21.6"E
60	*Anopheles dravidicus*	5169.4	OL742854	Larvae dipper	Kanchanaburi	Western	14°14'30.4"N 99°20'21.6"E
61	*Anopheles dravidicus*	5169.5	OL742855	Larvae dipper	Kanchanaburi	Western	14°14'30.4"N 99°20'21.6"E
62	*Anopheles dravidicus*	5169.6	OL742856	Larvae dipper	Kanchanaburi	Western	14°14'30.4"N 99°20'21.6"E
63	*Anopheles epiroticus*	1647	OL742857	Adult mosquito trapping	Trat	Eastern	12°13'54.7"N 102°34'47.0"E
64	*Anopheles epiroticus*	7234.1	OL742858	Adult mosquito trapping	Phang Nga	Southern	8°28'09.0"N 98°35'15.4"E
65	*Anopheles epiroticus*	7062	OL742859	Adult mosquito trapping	Surat Thani	Southern	9°13'56.9"N 99°14'15.3"E
66	*Anopheles epiroticus*	7084	OL742860	Adult mosquito trapping	Nakhon Si Thammarat	Southern	8°27'30.8"N 100°00'11.9"E
67	*Anopheles epiroticus*	7133	OL742861	Adult mosquito trapping	Krabi	Southern	8°04'18.2"N 98°57'42.7"E
68	*Anopheles harrisoni*	5594.1	OL742862	Larvae dipper	Kanchanaburi	Western	14°20'11.8"N 98°59'21.0"E
69	*Anopheles harrisoni*	5594.2	OL742863	Larvae dipper	Kanchanaburi	Western	14°20'11.8"N 98°59'21.0"E
70	*Anopheles harrisoni*	6934.4	OL742864	Larvae dipper	Kanchanaburi	Western	14°20'11.8"N 98°59'21.0"E
71	*Anopheles jamesii*	5634	OL742865	Adult mosquito trapping	Trat	Eastern	12°36'49.6"N 102°28'37.5"E
72	*Anopheles jamesii*	7140	OL742866	Adult mosquito trapping	Phang Nga	Southern	8°24'34.6"N 98°34'58.6"E
73	*Anopheles jamesii*	7177	OL742867	Adult mosquito trapping	Phang Nga	Southern	8°24'34.6"N 98°34'58.6"E
74	*Anopheles jamesii*	4098	OL742868	Adult mosquito trapping	Krabi	Southern	8°02'57.4"N 99°05'25.1"E

	Species	Specimen voucher	GenBank accession numbers	Collection	Province	Geographical region	Latitude/ longitude
75	*Anopheles maculatus*	5919.1	OL742869	Adult mosquito trapping	Trat	Eastern	12°36'49.6"N 102°28'37.5"E
76	*Anopheles maculatus*	5919.2	OL742870	Adult mosquito trapping	Trat	Eastern	12°36'49.6"N 102°28'37.5"E
77	*Anopheles maculatus*	5991	OL742871	Adult mosquito trapping	Trat	Eastern	12°12'59.6"N 102°39'00.8"E
78	*Anopheles maculatus*	6511TM	OL742872	Adult mosquito trapping	Tak	Western	17°33'26.2"N 97°55'15.8"E
79	*Anopheles maculatus*	7415TM	OL742873	Adult mosquito trapping	Tak	Western	17°33'26.2"N 97°55'15.8"E
80	*Anopheles minimus*	3482.1	OL742874	Adult mosquito trapping	Kanchanaburi	Western	14°07'06.2"N 99°01'16.2"E
81	*Anopheles minimus*	4322.1	OL742875	Adult mosquito trapping	Kanchanaburi	Western	14°07'06.2"N 99°01'16.2"E
82	*Anopheles minimus*	4040.4	OL742876	Adult mosquito trapping	Chachoengsao	Eastern	13°30'16.7"N 101°47'09.2"E
83	*Anopheles minimus*	7233	OL742877	Adult mosquito trapping	Phang Nga	Southern	8°28'09.0"N 98°35'15.4"E
84	*Anopheles minimus*	7247	OL742878	Adult mosquito trapping	Phang Nga	Southern	8°28'09.0"N 98°35'15.4"E
85	*Anopheles nemophilous*	3257	OL742879	Adult mosquito trapping	Ratchaburi	Western	13°22'33.2"N 99°16'25.9"E
86	*Anopheles nigerrimus*	5210.2	OL742880	Adult mosquito trapping	Narathiwat	Southern	6°21'19.3"N 101°53'41.6"E
87	*Anopheles nitidus*	5206	OL742881	Adult mosquito trapping	Narathiwat	Southern	6°21'19.3"N 101°53'41.6"E
88	*Anopheles nitidus*	5206.1	OL742882	Adult mosquito trapping	Narathiwat	Southern	6°21'19.3"N 101°53'41.6"E
89	*Anopheles nitidus*	5206.3	OL742883	Adult mosquito trapping	Narathiwat	Southern	6°21'19.3"N 101°53'41.6"E
90	*Anopheles nivipes*	5127	OL742884	Larvae dipper	Chachoengsao	Eastern	13°30'16.7"N 101°47'09.2"E
91	*Anopheles nivipes*	6323.1	OL742885	Larvae dipper	Ubon Ratchathani	Northeastern	14°25'12.6"N 105°11'19.0"E
92	*Anopheles paraliae*	3299	OL742886	Adult mosquito trapping	Ratchaburi	Western	13°21'35.3"N 99°15'05.0"E
93	*Anopheles paraliae*	3299.1	OL742887	Adult mosquito trapping	Ratchaburi	Western	13°21'35.3"N 99°15'05.0"E
94	*Anopheles paraliae*	3631	OL742888	Adult mosquito trapping	Ratchaburi	Western	13°21'51.4"N 99°15'41.9"E
95	*Anopheles paraliae*	3666	OL742889	Adult mosquito trapping	Ratchaburi	Western	13°21'51.4"N 99°15'41.9"E
96	*Anopheles paraliae*	1896.1	OL742890	Adult mosquito trapping	Samut Songkhram	Central	13°23'18.8"N 99°55'35.4"E
97	*Anopheles paraliae*	1897.2	OL742891	Adult mosquito trapping	Samut Songkhram	Central	13°23'18.8"N 99°55'35.4"E
98	*Anopheles peditaeniatus*	4488	OL742892	Adult mosquito trapping	Ratchaburi	Western	13°21'35.1"N 99°15'04.5"E
99	*Anopheles philippinensis*	6323.5	OL742893	Larvae dipper	Ubon Ratchathani	Northeastern	14°25'12.6"N 105°11'19.0"E
100	*Anopheles philippinensis*	6323.6	OL742894	Larvae dipper	Ubon Ratchathani	Northeastern	14°25'12.6"N 105°11'19.0"E
101	*Anopheles philippinensis*	5211	OL742895	Larvae dipper	Narathiwat	Southern	6°21'19.3"N 101°53'41.6"E
102	*Anopheles philippinensis*	5651	OL742896	Adult mosquito trapping	Trat	Eastern	12°36'49.6"N 102°28'37.5"E
103	*Anopheles pseudojamesi*	6471	OL742897	Adult mosquito trapping	Chiang Mai	Northern	18°51'18.5"N 98°42'07.3"E
104	*Anopheles pseudojamesi*	5127.1	OL742898	Larvae dipper	Chachoengsao	Eastern	13°30'16.7"N 101°47'09.2"E
105	*Anopheles pseudojamesi*	5615	OL742899	Adult mosquito trapping	Trat	Eastern	12°36'49.6"N 102°28'37.5"E
106	*Anopheles pseudojamesi*	5695	OL742900	Adult mosquito trapping	Trat	Eastern	12°36'49.6"N 102°28'37.5"E
107	*Anopheles pseudojamesi*	4152.4	OL742901	Larvae dipper	Narathiwat	Southern	6°21'19.3"N 101°53'41.6"E
108	*Anopheles pseudojamesi*	4152.5	OL742902	Larvae dipper	Narathiwat	Southern	6°21'19.3"N 101°53'41.6"E
109	*Anopheles pseudowillmori*	10575TM	OL742903	Larvae dipper	Tak	Western	17°33'26.2"N 97°55'15.8"E
110	*Anopheles pseudowillmori*	10974TM	OL742904	Larvae dipper	Tak	Western	17°33'26.2"N 97°55'15.8"E
111	*Anopheles pursati*	1884.2	OL742905	Adult mosquito trapping	Samut Songkhram	Central	13°23'18.8"N 99°55'35.4"E
112	*Anopheles pursati*	1897	OL742906	Adult mosquito trapping	Samut Songkhram	Central	13°23'18.8"N 99°55'35.4"E

	Species	Specimen voucher	GenBank accession numbers	Collection	Province	Geographical region	Latitude/ longitude
113	*Anopheles pursati*	1897.1	OL742907	Adult mosquito trapping	Samut Songkhram	Central	13°23'18.8"N 99°55'35.4"E
114	*Anopheles saeungae*	6121	OL742908	Adult mosquito trapping	Ubon Ratchathani	Northeastern	14°26'54.1"N 105°12'33.0"E
115	*Anopheles saeungae*	2941.1.1	OL742910	Adult mosquito trapping	Nakhon Si Thammarat	Southern	8°04'34.0"N 99°46'29.3"E
116	*Anopheles sawadwongporni*	4064TM	OL742914	Adult mosquito trapping	Tak	Western	17°33'26.2"N 97°55'15.8"E
117	*Anopheles sawadwongporni*	4110TM	OL742915	Adult mosquito trapping	Tak	Western	17°33'26.2"N 97°55'15.8"E
118	*Anopheles sawadwongporni*	4132TM	OL742916	Adult mosquito trapping	Tak	Western	17°33'26.2"N 97°55'15.8"E
119	*Anopheles sawadwongporni*	4565.3	OL742917	Adult mosquito trapping	Ratchaburi	Western	13°21'35.1"N 99°15'04.5"E
120	*Anopheles sawadwongporni*	4565.4	OL742918	Adult mosquito trapping	Ratchaburi	Western	13°21'35.1"N 99°15'04.5"E
121	*Anopheles sawadwongporni*	4874	OL742919	Larvae dipper	Ratchaburi	Western	13°21'28.7"N 99°15'05.7"E
122	*Anopheles sinensis*	4558	OL742920	Adult mosquito trapping	Ratchaburi	Western	13°21'35.1"N 99°15'04.5"E
123	*Anopheles sinensis*	5096.1	OL742921	Larvae dipper	Chaiyaphum	Northeastern	16°25'16.2"N 101°57'46.8"E
124	*Anopheles subpictus*	4877.2	OL742922	Larvae dipper	Ratchaburi	Western	13°21'28.7"N 99°15'05.7"E
125	*Anopheles subpictus*	2956.2	OL742923	Adult mosquito trapping	Nakhon Pathom	Central	13°53'29.6"N 100°00'08.8"E
126	*Anopheles subpictus*	5124	OL742924	Larvae dipper	Chachoengsao	Eastern	13°27'16.6"N 101°38'37.2"E
127	*Anopheles subpictus*	5094.2	OL742925	Larvae dipper	Chaiyaphum	Northeastern	16°25'16.2"N 101°57'46.8"E
128	*Anopheles tessellatus*	4442.1	OL742926	Adult mosquito trapping	Nan	Northern	18°43'11.0"N 100°46'04.0"E
129	*Anopheles tessellatus*	4442.2	OL742927	Adult mosquito trapping	Nan	Northern	18°43'11.0"N 100°46'04.0"E
130	*Anopheles tessellatus*	4443	OL742928	Adult mosquito trapping	Nan	Northern	18°43'11.0"N 100°46'04.0"E
131	*Anopheles tessellatus*	4443.1	OL742929	Adult mosquito trapping	Nan	Northern	18°43'11.0"N 100°46'04.0"E
132	*Anopheles tessellatus*	7165	OL742930	Adult mosquito trapping	Phang Nga	Southern	8°24'34.6"N 98°34'58.6"E
133	*Anopheles vagus*	3599	OL742931	Adult mosquito trapping	Ratchaburi	Western	13°21'35.3"N 99°15'05.0"E
134	*Anopheles vagus*	3599.1	OL742932	Adult mosquito trapping	Ratchaburi	Western	13°21'35.3"N 99°15'05.0"E
135	*Anopheles vagus*	2956.1	OL742933	Adult mosquito trapping	Nakhon Pathom	Central	13°53'29.6"N 100°00'08.8"E
136	*Anopheles varuna*	4594	OL742934	Adult mosquito trapping	Kanchanaburi	Western	14°07'06.2"N 99°01'16.2"E
137	*Anopheles varuna*	4459	OL742935	Adult mosquito trapping	Ratchaburi	Western	13°21'35.1"N 99°15'04.5"E
138	*Anopheles wejchoochotei*	4324.3	OL742936	Adult mosquito trapping	Kanchanaburi	Western	14°07'06.2"N 99°01'16.2"E
139	*Anopheles wejchoochotei*	4425	OL742937	Adult mosquito trapping	Kanchanaburi	Western	14°07'06.2"N 99°01'16.2"E
140	*Anopheles wejchoochotei*	1896	OL742938	Adult mosquito trapping	Samut Songkhram	Central	13°23'18.8"N 99°55'35.4"E
141	*Anopheles wejchoochotei*	1896.2	OL742939	Adult mosquito trapping	Samut Songkhram	Central	13°23'18.8"N 99°55'35.4"E
142	*Anopheles wejchoochotei*	4049.1	OL742940	Adult mosquito trapping	Chachoengsao	Eastern	13°30'16.7"N 101°47'09.2"E
143	*Anopheles wejchoochotei*	4049.2	OL742941	Adult mosquito trapping	Chachoengsao	Eastern	13°30'16.7"N 101°47'09.2"E
144	*Anopheles wejchoochotei*	5326.1	OL742942	Adult mosquito trapping	Chachoengsao	Eastern	13°30'16.7"N 101°47'09.2"E
145	*Anopheles wejchoochotei*	5326.2	OL742943	Adult mosquito trapping	Chachoengsao	Eastern	13°30'16.7"N 101°47'09.2"E
146	*Armigeres subalbatus*	6601.1	OL742944	Larvae dipper	Chiang Mai	Northern	18°51'18.5"N 98°42'07.3"E
147	*Armigeres subalbatus*	7652.1	OL742945	Adult mosquito trapping	Kanchanaburi	Western	14°07'06.2"N 99°01'16.2"E
148	*Armigeres subalbatus*	6306.1	OL742946	Adult mosquito trapping	Ubon Ratchathani	Northeastern	14°33'36.3"N 105°21'51.5"E
149	*Armigeres subalbatus*	5285.1	OL742947	Adult mosquito trapping	Chanthaburi	Eastern	12°51'29.8"N 102°16'32.0"E
150	*Armigeres subalbatus*	7221.1	OL742948	Adult mosquito trapping	Phang Nga	Southern	8°28'09.0"N 98°35'15.4"E

	Species	Specimen voucher	GenBank accession numbers	Collection	Province	Geographical region	Latitude/ longitude
151	*Armigeres durhami*	6555.1	OL742949	Adult mosquito trapping	Chiang Mai	Northern	18°51'18.5"N 98°42'07.3"E
152	*Armigeres durhami*	6596.1	OL742950	Adult mosquito trapping	Chiang Mai	Northern	18°51'18.5"N 98°42'07.3"E
153	*Armigeres durhami*	6596.2	OL742951	Adult mosquito trapping	Chiang Mai	Northern	18°51'18.5"N 98°42'07.3"E
154	*Armigeres durhami*	6596.3	OL742952	Adult mosquito trapping	Chiang Mai	Northern	18°51'18.5"N 98°42'07.3"E
155	*Armigeres durhami*	6596.4	OL742953	Adult mosquito trapping	Chiang Mai	Northern	18°51'18.5"N 98°42'07.3"E
156	*Armigeres durhami*	6596.5	OL742954	Adult mosquito trapping	Chiang Mai	Northern	18°51'18.5"N 98°42'07.3"E
157	*Armigeres flavus*	7738	OL742955	Adult mosquito trapping	Ratchaburi	Western	13°22'35.7"N 99°16'37.2"E
158	*Armigeres flavus*	7783.4	OL742956	Adult mosquito trapping	Ratchaburi	Western	13°22'35.7"N 99°16'37.2"E
159	*Armigeres malayi*	3850.1	OL742957	Adult mosquito trapping	Ratchaburi	Western	13°22'37.2"N 99°15'17.2"E
160	*Collessius macfarlanei*	6334.1	OL743102	Larvae dipper	Ubon Ratchathani	Northeastern	14°26'54.1"N 105°12'33.0"E
161	*Collessius macfarlanei*	6334.2	OL743103	Larvae dipper	Ubon Ratchathani	Northeastern	14°26'54.1"N 105°12'33.0"E
162	*Collessius macfarlanei*	6334.3	OL743104	Larvae dipper	Ubon Ratchathani	Northeastern	14°26'54.1"N 105°12'33.0"E
163	*Collessius macfarlanei*	6334.4	OL743105	Larvae dipper	Ubon Ratchathani	Northeastern	14°26'54.1"N 105°12'33.0"E
164	*Coquillettidia crassipes*	6567.1	OL742958	Adult mosquito trapping	Chiang Mai	Northern	19°10'26.4"N 99°01'49.2"E
165	*Coquillettidia crassipes*	5933.1	OL742959	Adult mosquito trapping	Trat	Eastern	12°12'59.6"N 102°39'00.8"E
166	*Coquillettidia crassipes*	6166.1	OL742960	Adult mosquito trapping	Ubon Ratchathani	Northeastern	14°26'54.1"N 105°12'33.0"E
167	*Coquillettidia crassipes*	7209.1	OL742961	Adult mosquito trapping	Phang Nga	Southern	8°28'09.0"N 98°35'15.4"E
168	*Coquillettidia ochracea*	5306	OL742962	Adult mosquito trapping	Chanthaburi	Eastern	12°32'10.5"N 102°05'30.1"E
169	*Coquillettidia ochracea*	4174.1	OL742963	Adult mosquito trapping	Narathiwat	Southern	6°21'19.3"N 101°53'41.6"E
170	*Coquillettidia ochracea*	4174.2	OL742964	Adult mosquito trapping	Narathiwat	Southern	6°21'19.3"N 101°53'41.6"E
171	*Coquillettidia ochracea*	4174.3	OL742965	Adult mosquito trapping	Narathiwat	Southern	6°21'19.3"N 101°53'41.6"E
172	*Coquillettidia ochracea*	4174.4	OL742966	Adult mosquito trapping	Narathiwat	Southern	6°21'19.3"N 101°53'41.6"E
173	*Coquillettidia ochracea*	4175.1	OL742967	Adult mosquito trapping	Narathiwat	Southern	6°21'19.3"N 101°53'41.6"E
174	*Culex bicornutus*	5164.1	OL742968	Larvae dipper	Kanchanaburi	Western	14°14'30.4"N 99°20'21.6"E
175	*Culex bicornutus*	6936.1	OL742969	Larvae dipper	Kanchanaburi	Western	14°14'30.4"N 99°20'21.6"E
176	*Culex bicornutus*	4584.1	OL742970	Larvae dipper	Ratchaburi	Western	13°28'36.2"N 99°14'34.1"E
177	*Culex bicornutus*	4584.2	OL742971	Larvae dipper	Ratchaburi	Western	13°28'36.2"N 99°14'34.1"E
178	*Culex bicornutus*	4584.3	OL742972	Larvae dipper	Ratchaburi	Western	13°28'36.2"N 99°14'34.1"E
179	*Culex bicornutus*	4584.4	OL742973	Larvae dipper	Ratchaburi	Western	13°28'36.2"N 99°14'34.1"E
180	*Culex bicornutus*	5449.1	OL742974	Larvae dipper	Ratchaburi	Western	13°22'33.7"N 99°16'25.9"E
181	*Culex bicornutus*	5449.2	OL742975	Larvae dipper	Ratchaburi	Western	13°22'33.7"N 99°16'25.9"E
182	*Culex bitaeniorhynchus*	6505	OL742976	Adult mosquito trapping	Mae Hong Son	Northern	18°07'36.7"N 97°57'03.4"E
183	*Culex bitaeniorhynchus*	6505.1	OL742977	Adult mosquito trapping	Mae Hong Son	Northern	18°07'36.7"N 97°57'03.4"E
184	*Culex bitaeniorhynchus*	4864.1	OL742978	Larvae dipper	Ratchaburi	Western	13°27'46.7"N 99°15'11.9"E
185	*Culex bitaeniorhynchus*	6699.1	OL742979	Adult mosquito trapping	Ratchaburi	Western	13°22'35.7"N 99°16'37.2"E
186	*Culex brevipalpis*	7677.1	OL742980	Larvae dipper	Kanchanaburi	Western	14°14'30.3"N 99°20'21.5"E
187	*Culex brevipalpis*	5444.1	OL742981	Larvae dipper	Ratchaburi	Western	13°22'48.0"N 99°16'31.7"E
188	*Culex brevipalpis*	6792.1	OL742982	Larvae dipper	Ratchaburi	Western	13°22'35.7"N 99°16'37.2"E

	Species	Specimen voucher	GenBank accession numbers	Collection	Province	Geographical region	Latitude/ longitude
189	*Culex brevipalpis*	6316.2	OL742983	Adult mosquito trapping	Ubon Ratchathani	Northeastern	14°26'54.1"N 105°12'33.0"E
190	*Culex epidesmus*	1448.1	OL742984	Adult mosquito trapping	Tak	Western	17°33'26.2"N 97°55'15.8"E
191	*Culex epidesmus*	1448.2	OL742985	Adult mosquito trapping	Tak	Western	17°33'26.2"N 97°55'15.8"E
192	*Culex epidesmus*	1448.3	OL742986	Adult mosquito trapping	Tak	Western	17°33'26.2"N 97°55'15.8"E
193	*Culex fuscocephala*	6529	OL742987	Adult mosquito trapping	Mae Hong Son	Northern	18°07'36.7"N 97°57'03.4"E
194	*Culex fuscocephala*	6425.9	OL742988	Adult mosquito trapping	Chiang Mai	Northern	18°41'33.9"N 98°55'48.9"E
195	*Culex fuscocephala*	6178	OL742989	Adult mosquito trapping	Ubon Ratchathani	Northeastern	14°26'54.1"N 105°12'33.0"E
196	*Culex fuscocephala*	7222.1	OL742990	Adult mosquito trapping	Phang Nga	Southern	8°28'09.0"N 98°35'15.4"E
197	*Culex fuscocephala*	7222.2	OL742991	Adult mosquito trapping	Phang Nga	Southern	8°28'09.0"N 98°35'15.4"E
198	*Culex gelidus*	6545	OL742992	Adult mosquito trapping	Chiang Mai	Northern	18°51'18.5"N 98°42'07.3"E
199	*Culex gelidus*	4281.2	OL742993	Adult mosquito trapping	Ratchaburi	Western	13°21'35.1"N 99°15'04.5"E
200	*Culex gelidus*	1905.1	OL742994	Adult mosquito trapping	Samut Songkhram	Central	13°23'18.8"N 99°55'35.4"E
201	*Culex gelidus*	4016.1	OL742995	Adult mosquito trapping	Chachoengsao	Eastern	13°30'16.7"N 101°47'09.2"E
202	*Culex gelidus*	6256.1	OL742996	Adult mosquito trapping	Ubon Ratchathani	Northeastern	14°33'36.3"N 105°21'51.5"E
203	*Culex gelidus*	7205.1	OL742997	Adult mosquito trapping	Phang Nga	Southern	8°28'09.0"N 98°35'15.4"E
204	*Culex infantulus*	7335.1	OL742998	Larvae dipper	Kanchanaburi	Western	14°08'26.9"N 98°59'43.7"E
205	*Culex infantulus*	7335.2	OL742999	Larvae dipper	Kanchanaburi	Western	14°08'26.9"N 98°59'43.7"E
206	*Culex infantulus*	7335.3	OL742300	Larvae dipper	Kanchanaburi	Western	14°08'26.9"N 98°59'43.7"E
207	*Culex infantulus*	7335.4	OL743001	Adult mosquito trapping	Kanchanaburi	Western	14°07'06.2"N 99°01'16.2"E
208	*Culex infantulus*	4583.1	OL743002	Larvae dipper	Ratchaburi	Western	13°28'36.2"N 99°14'34.1"E
209	*Culex infantulus*	4583.2	OL743003	Larvae dipper	Ratchaburi	Western	13°28'36.2"N 99°14'34.1"E
210	*Culex infantulus*	4583.3	OL743004	Larvae dipper	Ratchaburi	Western	13°28'36.2"N 99°14'34.1"E
211	*Culex infantulus*	4583.4	OL743005	Larvae dipper	Ratchaburi	Western	13°28'36.2"N 99°14'34.1"E
212	*Culex murrelli*	6605.1	OL743006	Larvae dipper	Chiang Mai	Northern	18°49'59.1"N 98°34'35.0"E
213	*Culex murrelli*	4854.1	OL743007	Larvae dipper	Ratchaburi	Western	13°31'33.0"N 99°14'23.7"E
214	*Culex murrelli*	5320.1	OL743008	Larvae dipper	Chanthaburi	Eastern	12°51'29.8"N 102°16'32.0"E
215	*Culex murrelli*	1697	OL743009	Larvae dipper	Trat	Eastern	11°40'46.3"N 102°32'41.8"E
216	*Culex murrelli*	3247.1	OL743010	Adult mosquito trapping	Surat Thani	Southern	9°04'50.1"N 99°36'14.5"E
217	*Culex murrelli*	3247.2	OL743011	Adult mosquito trapping	Surat Thani	Southern	9°04'50.1"N 99°36'14.5"E
218	*Culex nigropunctatus*	6394.1	OL743012	Adult mosquito trapping	Chiang Mai	Northern	18°41'33.9"N 98°55'48.9"E
219	*Culex nigropunctatus*	4907.2	OL743013	Adult mosquito trapping	Kanchanaburi	Western	14°07'06.2"N 99°01'16.2"E
220	*Culex nigropunctatus*	7443.1	OL743014	Adult mosquito trapping	Ratchaburi	Western	13°22'37.2"N 99°15'17.3"E
221	*Culex nigropunctatus*	5134.1	OL743015	Larvae dipper	Phetchaburi	Western	12°42'11.9"N 99°38'45.5"E
222	*Culex nigropunctatus*	5946.1	OL743016	Adult mosquito trapping	Trat	Eastern	12°20'40.5"N 102°26'58.8"E
223	*Culex nigropunctatus*	6074.1	OL743017	Adult mosquito trapping	Nakhon Ratchasima	Northeastern	14°30'56.4"N 101°22'31.7"E
224	*Culex nigropunctatus*	6282.1	OL743018	Adult mosquito trapping	Ubon Ratchathani	Northeastern	14°33'36.3"N 105°21'51.5"E
225	*Culex pallidothorax*	1383	OL743019	Adult mosquito trapping	Tak	Western	17°33'26.2"N 97°55'15.8"E
226	*Culex pallidothorax*	1385	OL743020	Adult mosquito trapping	Tak	Western	17°33'26.2"N 97°55'15.8"E

	Species	Specimen voucher	GenBank accession numbers	Collection	Province	Geographical region	Latitude/ longitude
227	*Culex pallidothorax*	1386	OL743021	Adult mosquito trapping	Tak	Western	17°33'26.2"N 97°55'15.8"E
228	*Culex pallidothorax*	6936.2	OL743022	Larvae dipper	Kanchanaburi	Western	14°14'30.4"N 99°20'21.6"E
229	*Culex pallidothorax*	6936.3	OL743023	Larvae dipper	Kanchanaburi	Western	14°14'30.4"N 99°20'21.6"E
230	*Culex pallidothorax*	6936.4	OL743024	Larvae dipper	Kanchanaburi	Western	14°14'30.4"N 99°20'21.6"E
231	*Culex pallidothorax*	6316.1	OL743025	Adult mosquito trapping	Ubon Ratchathani	Northeastern	14°26'54.1"N 105°12'33.0"E
232	*Culex pseudovishnui*	4162.1	OL743028	Adult mosquito trapping	Narathiwat	Southern	6°21'19.3"N 101°53'41.6"E
233	*Culex pseudovishnui*	4181.1	OL743029	Adult mosquito trapping	Narathiwat	Southern	6°21'19.3"N 101°53'41.6"E
234	*Culex quinquefasciatus*	6595.1	OL743030	Larvae dipper	Chiang Mai	Northern	18°51'18.4"N 98°42'07.5"E
235	*Culex quinquefasciatus*	4778.1	OL743031	Adult mosquito trapping	Kanchanaburi	Western	14°07'06.2"N 99°01'16.2"E
236	*Culex quinquefasciatus*	5378	OL743032	Adult mosquito trapping	Ratchaburi	Western	13°22'42.0"N 99°16'30.8"E
237	*Culex quinquefasciatus*	3109.1	OL743033	Adult mosquito trapping	Nakhon Pathom	Central	13°53'29.6"N 100°00'08.8"E
238	*Culex quinquefasciatus*	6033	OL743034	Adult mosquito trapping	Trat	Eastern	12°33'14.3"N 102°32'43.6"E
239	*Culex quinquefasciatus*	6033.1	OL743035	Adult mosquito trapping	Trat	Eastern	12°33'14.3"N 102°32'43.6"E
240	*Culex quinquefasciatus*	6230.8	OL743036	Adult mosquito trapping	Ubon Ratchathani	Northeastern	14°26'54.1"N 105°12'33.0"E
241	*Culex quinquefasciatus*	7086.1	OL743037	Adult mosquito trapping	Nakhon Si Thammarat	Southern	8°27'30.8"N 100°00'11.9"E
242	*Culex sitiens*	4072.1	OL743038	Adult mosquito trapping	Samut Songkhram	Central	13°22'08.8"N 99°53'23.8"E
243	*Culex sitiens*	6023.1	OL743039	Adult mosquito trapping	Trat	Eastern	12°13'54.7"N 102°34'47.0"E
244	*Culex sitiens*	7150.1	OL743040	Adult mosquito trapping	Phang Nga	Southern	8°24'34.6"N 98°34'58.6"E
245	*Culex sitiens*	7014.1	OL743041	Adult mosquito trapping	Surat Thani	Southern	9°13'56.9"N 99°14'15.3"E
246	*Culex sitiens*	7019	OL743042	Adult mosquito trapping	Surat Thani	Southern	9°13'56.9"N 99°14'15.3"E
247	*Culex sitiens*	7108.1	OL743043	Adult mosquito trapping	Krabi	Southern	8°04'18.2"N 98°57'42.7"E
248	*Culex sitiens*	7108.2	OL743044	Adult mosquito trapping	Krabi	Southern	8°04'18.2"N 98°57'42.7"E
249	*Culex tritaeniorhynchus*	6429.9	OL743045	Adult mosquito trapping	Chiang Mai	Northern	18°41'33.9"N 98°55'48.9"E
250	*Culex tritaeniorhynchus*	6431.3	OL743046	Adult mosquito trapping	Chiang Mai	Northern	18°41'33.9"N 98°55'48.9"E
251	*Culex tritaeniorhynchus*	3263.1	OL743047	Adult mosquito trapping	Ratchaburi	Western	13°22'33.2"N 99°16'25.9"E
252	*Culex tritaeniorhynchus*	3162.1	OL743048	Adult mosquito trapping	Samut Songkhram	Central	13°23'18.8"N 99°55'35.4"E
253	*Culex tritaeniorhynchus*	5316.1	OL743049	Larvae dipper	Chanthaburi	Eastern	12°51'29.8"N 102°16'32.0"E
254	*Culex tritaeniorhynchus*	6216.2	OL743050	Adult mosquito trapping	Ubon Ratchathani	Northeastern	14°26'54.1"N 105°12'33.0"E
255	*Culex vishnui*	6440.1	OL743051	Larvae dipper	Chiang Mai	Northern	18°41'33.9"N 98°55'48.9"E
256	*Culex vishnui*	4602	OL743052	Adult mosquito trapping	Kanchanaburi	Western	14°07'06.2"N 99°01'16.2"E
257	*Culex vishnui*	7645.1	OL743053	Adult mosquito trapping	Kanchanaburi	Western	14°07'06.2"N 99°01'16.2"E
258	*Culex vishnui*	6321.1	OL743054	Adult mosquito trapping	Ubon Ratchathani	Northeastern	14°26'54.1"N 105°12'33.0"E
259	*Culex vishnui*	6331.2	OL743055	Larvae dipper	Ubon Ratchathani	Northeastern	14°29'12.6"N 105°12'55.7"E
260	*Culex vishnui*	6331.3	OL743056	Larvae dipper	Ubon Ratchathani	Northeastern	14°29'12.6"N 105°12'55.7"E
261	*Culex vishnui*	6335	OL743057	Adult mosquito trapping	Ubon Ratchathani	Northeastern	14°26'54.1"N 105°12'33.0"E
262	*Lutzia vorax*	6292	OL743058	Adult mosquito trapping	Ubon Ratchathani	Northeastern	14°33'36.3"N 105°21'51.5"E
263	*Lutzia vorax*	4903.1	OL743059	Larvae dipper	Kanchanaburi	Western	14°14'30.4"N 99°20'21.6"E
264	*Lutzia fuscana*	5447.1	OL743060	Larvae dipper	Ratchaburi	Western	13°22'33.7"N 99°16'25.9"E

	Species	Specimen voucher	GenBank accession numbers	Collection	Province	Geographical region	Latitude/ longitude
265	*Lutzia fuscana*	5447.2	OL743061	Larvae dipper	Ratchaburi	Western	13°22'33.7"N 99°16'25.9"E
266	*Lutzia fuscana*	5447.3	OL743062	Larvae dipper	Ratchaburi	Western	13°22'33.7"N 99°16'25.9"E
267	*Lutzia fuscana*	5102.1	OL743063	Larvae dipper	Chachoengsao	Eastern	13°27'15.3"N 101°46'26.4"E
268	*Lutzia fuscana*	5102.2	OL743064	Larvae dipper	Chachoengsao	Eastern	13°27'15.3"N 101°46'26.4"E
269	*Lutzia fuscana*	5102.3	OL743065	Larvae dipper	Chachoengsao	Eastern	13°27'15.3"N 101°46'26.4"E
270	*Lutzia chiangmaiensis*	6318	OL743066	Adult mosquito trapping	Ubon Ratchathani	Northeastern	14°26'54.1"N 105°12'33.0"E
271	*Lutzia chiangmaiensis*	5599	OL743067	Larvae dipper	Ratchaburi	Western	13°22'35.7"N 99°16'37.2"E
272	*Lutzia chiangmaiensis*	5446.1	OL743068	Larvae dipper	Ratchaburi	Western	13°22'35.7"N 99°16'37.2"E
273	*Lutzia chiangmaiensis*	5432.1	OL743069	Larvae dipper	Ratchaburi	Western	13°22'35.7"N 99°16'37.2"E
274	*Lutzia chiangmaiensis*	4437.1	OL743070	Adult mosquito trapping	Kanchanaburi	Western	14°06'37.5"N 98°59'52.9"E
275	*Lutzia chiangmaiensis*	5129	OL743071	Adult mosquito trapping	Chachoengsao	Eastern	13°30'16.7"N 101°47'09.2"E
276	*Mansonia annulifera*	6564.1	OL743072	Adult mosquito trapping	Chiang Mai	Northern	19°10'26.4"N 99°01'49.2"E
277	*Mansonia annulifera*	4486	OL743073	Adult mosquito trapping	Ratchaburi	Western	13°21'35.1"N 99°15'04.5"E
278	*Mansonia annulifera*	3558.1	OL743074	Adult mosquito trapping	Samut Songkhram	Central	13°22'08.8"N 99°53'23.8"E
279	*Mansonia annulifera*	5831.1	OL743075	Adult mosquito trapping	Trat	Eastern	11°54'00.5"N 102°48'42.8"E
280	*Mansonia annulifera*	6079.1	OL743076	Adult mosquito trapping	Nakhon Ratchasima	Northeastern	14°30'56.4"N 101°22'31.7"E
281	*Mansonia annulifera*	4197.1	OL743077	Adult mosquito trapping	Narathiwat	Southern	6°21'19.3"N 101°53'41.6"E
282	*Mansonia bonneae*	4185.1	OL743078	Adult mosquito trapping	Narathiwat	Southern	6°21'19.3"N 101°53'41.6"E
283	*Mansonia bonneae*	4185.2	OL743079	Adult mosquito trapping	Narathiwat	Southern	6°21'19.3"N 101°53'41.6"E
284	*Mansonia bonneae*	4185.3	OL743080	Adult mosquito trapping	Narathiwat	Southern	6°21'19.3"N 101°53'41.6"E
285	*Mansonia bonneae*	4185.4	OL743081	Adult mosquito trapping	Narathiwat	Southern	6°21'19.3"N 101°53'41.6"E
286	*Mansonia bonneae*	4189.1	OL743082	Adult mosquito trapping	Narathiwat	Southern	6°21'19.3"N 101°53'41.6"E
287	*Mansonia dives*	1155	OL743083	Adult mosquito trapping	Tak	Western	17°33'26.2"N 97°55'15.8"E
288	*Mansonia dives*	1156	OL743084	Adult mosquito trapping	Tak	Western	17°33'26.2"N 97°55'15.8"E
289	*Mansonia indiana*	3942	OL743087	Adult mosquito trapping	Kanchanaburi	Western	14°06'37.5"N 98°59'52.9"E
290	*Mansonia indiana*	3973	OL743088	Adult mosquito trapping	Kanchanaburi	Western	14°06'37.5"N 98°59'52.9"E
291	*Mansonia indiana*	4268	OL743089	Adult mosquito trapping	Ratchaburi	Western	13°22'37.2"N 99°15'17.2"E
292	*Mansonia uniformis*	6423.1	OL743090	Adult mosquito trapping	Chiang Mai	Northern	18°41'33.9"N 98°55'48.9"E
293	*Mansonia uniformis*	7488.1	OL743091	Adult mosquito trapping	Ratchaburi	Western	13°22'35.7"N 99°16'37.2"E
294	*Mansonia uniformis*	3559.1	OL743092	Adult mosquito trapping	Samut Songkhram	Central	13°22'08.8"N 99°53'23.8"E
295	*Mansonia uniformis*	5921.1	OL743093	Adult mosquito trapping	Trat	Eastern	11°54'00.5"N 102°48'42.8"E
296	*Mansonia uniformis*	6236.1	OL743094	Adult mosquito trapping	Ubon Ratchathani	Northeastern	14°26'54.1"N 105°12'33.0"E
297	*Mansonia uniformis*	7021.1	OL743095	Adult mosquito trapping	Surat Thani	Southern	9°13'56.9"N 99°14'15.3"E
298	*Mimomyia aurea*	5215.1	OL743106	Adult mosquito trapping	Narathiwat	Southern	6°21'19.3"N 101°53'41.6"E
299	*Mimomyia aurea*	5215.3	OL743107	Adult mosquito trapping	Narathiwat	Southern	6°21'19.3"N 101°53'41.6"E
300	*Mimomyia aurea*	5215.4	OL743108	Adult mosquito trapping	Narathiwat	Southern	6°21'19.3"N 101°53'41.6"E
301	*Ochlerotatus vigilax*	7193.1	OL742822	Adult mosquito trapping	Phang Nga	Southern	8°24'34.6"N 98°34'58.6"E
302	*Ochlerotatus vigilax*	7193.2	OL742823	Adult mosquito trapping	Phang Nga	Southern	8°24'34.6"N 98°34'58.6"E

	Species	Specimen voucher	GenBank accession numbers	Collection	Province	Geographical region	Latitude/ longitude
303	*Ochlerotatus vigilax*	7193.3	OL742824	Adult mosquito trapping	Phang Nga	Southern	8°24'34.6"N 98°34'58.6"E
304	*Ochlerotatus vigilax*	7193.4	OL742825	Adult mosquito trapping	Phang Nga	Southern	8°24'34.6"N 98°34'58.6"E
305	*Rhinoskusea longirostris*	7531	OL743109	Adult mosquito trapping	Ratchaburi	Western	13°22'35.7"N 99°16'37.2"E
306	*Rhinoskusea longirostris*	7564	OL743110	Adult mosquito trapping	Ratchaburi	Western	13°22'35.7"N 99°16'37.2"E
307	*Toxorhynchites splendens*	3244	OL743111	Adult mosquito trapping	Surat Thani	Southern	9°04'50.1"N 99°36'14.5"E
308	*Toxorhynchites splendens*	3244.1	OL743112	Adult mosquito trapping	Surat Thani	Southern	9°04'50.1"N 99°36'14.5"E
309	*Uranotaenia obscura*	7785.1	OL743113	Larvae dipper	Ratchaburi	Western	13°22'35.7"N 99°16'37.2"E
310	*Uranotaenia obscura*	7785.2	OL743114	Larvae dipper	Ratchaburi	Western	13°22'35.7"N 99°16'37.2"E
